# PARP Inhibitor Maintenance After First-Line Chemotherapy in Advanced-Stage Epithelial Ovarian Cancer: A Systematic Review and Meta-Analysis

**DOI:** 10.1001/jamanetworkopen.2025.41648

**Published:** 2025-11-05

**Authors:** Stamatios Petousis, Ilker Kahramanoglu, Christian Appenzeller-Herzog, Martina A. Angeles, Chrysoula Margioula-Siarkou, Joanna Kacperczyk-Bartnik, Esra Bilir, Christos Chatzakis, Giuseppe Caruso, Nicolò Bizzarri, Konstantinos Dinas, David D.L. Bowtell, Dale W. Garsed, Isabelle Ray-Coquard, Jonathan A. Ledermann, Michael Friedlander, Alexandros Sotiriadis, Viola Heinzelmann-Schwarz, Tibor A. Zwimpfer

**Affiliations:** 1 Second Department of Obstetrics and Gynecology, Aristotle University of Thessaloniki, Thessaloniki, Greece; 2 Department of Gynaecologic Oncology, Biruni University, Istanbul, Turkey; 3 University Medical Library, University of Basel, Basel, Switzerland; 4 Gynaecologic Oncology Unit, Vall d’Hebron Barcelona Hospital Campus, Barcelona, Spain; 5 II Department of Obstetrics and Gynaecology, Medical University of Warsaw, Warsaw, Poland; 6 Department of Obstetrics and Gynaecology, St. Josefs-Hospital Wiesbaden Academic Teaching Hospital of Johannes Gutenberg, University of Mainz, Wiesbaden, Hessen, Germany; 7 Department of Gynaecologic Oncology, Koç University School of Medicine, Istanbul, Turkey; 8 Division of Gynecologic Oncology, European Institute of Oncology, Istituto di Ricovero e Cura a Carattere Scientifico, Milan, Italy; 9 Unità Operativa Complessa Ginecologia Oncologica, Dipartimento per la Salute Della Donna e del Bambino e Della Salute Pubblica, Policlinico Agostino Gemelli Istituto di Ricovero e Cura a Carattere Scientifico, Rome, Italy; 10 Peter MacCallum Cancer Centre, Melbourne, Australia; 11 Sir Peter MacCallum Department of Oncology, The University of Melbourne, Australia; 12 Centre Leon Berard and Université Claude Bernard, Lyon, France; 13 University College London Cancer Institute, London, United Kingdom; 14 University College London Hospitals, London, United Kingdom; 15 Prince of Wales Clinical School, University of New South Wales and Royal Hospital for Women, Randwick, Australia; 16 Gynaecological Cancer Centre, University Hospital Basel, Basel, Switzerland; 17 Ovarian Cancer Research, Department of Biomedicine, University of Basel, Basel, Switzerland

## Abstract

**Importance:**

First-line maintenance therapy with poly(adenosine diphosphate-ribose) polymerase inhibitors (PARP inhibitors) after platinum-based chemotherapy improves progression-free survival (PFS) in advanced epithelial ovarian cancer (EOC), particularly in patients with *BRCA*-variant or homologous recombination-deficient tumors. However, overall survival (OS) benefits remain uncertain, and toxic effect profiles emphasize the need for optimized patient and agent selection.

**Objective:**

To evaluate the efficacy and safety of first-line PARP inhibitor maintenance therapy compared with chemotherapy alone in advanced-stage EOC, with subgroup analyses by *BRCA* and HRD status, up-front or interval surgery, chemotherapy response, and residual disease.

**Data sources:**

Medline, Embase, Web of Science, Cochrane Central Register of Controlled Trials, and ClinicalTrials.gov were searched from database inception to August 19, 2024.

**Study Selection:**

Randomized clinical trials and prospective 2-arm studies evaluating PARP inhibitor maintenance therapy in patients with advanced-stage EOC responding to first-line platinum-based chemotherapy were included.

**Data Extraction and Synthesis:**

The Preferred Reporting Items for Systematic Reviews and Meta-Analyses (PRISMA) reporting guideline was followed in reporting this study. Data were independently extracted by 2 reviewers. Random-effects models were used for meta-analysis. Risk of bias was assessed with Cochrane risk of bias and certainty of evidence with the Grading of Recommendations Assessment, Development, and Evaluation (GRADE) framework.

**Main Outcomes and Measures:**

Primary outcomes were PFS and OS; secondary outcomes included high-grade adverse events.

**Results:**

A total of 7 randomized clinical trials with 4013 patients with advanced-stage epithelial ovarian cancer responding to first-line platinum-based chemotherapy were included. PARP inhibitor maintenance was associated with improved PFS in the overall population (hazard ratio [HR], 0.57; 95% CI, 0.46-0.70; high certainty) and in all molecular subgroups except the homologous recombination proficient group (*BRCA* variant: HR, 0.40; 95% CI, 0.35-0.45; *BRCA* wild type: HR, 0.62; 95% CI, 0.44-0.86; homologous recombination deficient: HR, 0.44; 95% CI, 0.39-0.50; all high certainty). PFS benefits were consistent across surgical timing, chemotherapy responses, and residual disease; for example, surgical timing had HRs of 0.51 (95% CI, 0.31-0.84) for neoadjuvant chemotherapy and 0.54 (95% CI, 0.36-0.81) for primary cytoreductive surgery (all high certainty). No molecular subgroup showed a statistically significant OS improvement (high to low certainty). High-grade adverse events were more common in the PARP inhibitor group (HR, 2.40; 95% CI, 1.16-4.93; high certainty). Observed treatment efficacy and toxic effects varied across PARP inhibitor regimens; for example, the risk ratio for any recurrence or death in the overall study group ranged from 0.53 (95% CI, 0.40-0.70) for senaparib to 0.83 (95% CI, 0.68-1.00) for olaparib, while the risk ratio for high-grade adverse events ranged from 1.15 (95% CI, 0.64-2.06) for veliparib to 4.73 (95% CI, 2.77-8.07) for niraparib.

**Conclusions and Relevance:**

In this study, no subgroup showed an association between first-line PARP inhibitor maintenance therapy in advanced-stage EOC and improved OS, and findings suggest that the consistency of associated PFS benefits may vary, particularly in homologous recombination proficient and *BRCA* wild type tumors. Variability in efficacy and toxic effects across subgroups and PARP inhibitor regimens underscores the importance of individualized treatment decisions.

## Key Points

**Question:**

What is the association of first-line poly(adenosine diphosphate-ribose) polymerase inhibitor (PARP inhibitor) maintenance therapy with survival outcomes in epithelial ovarian cancer, and how do outcomes vary across regimens and subgroups?

**Findings:**

In this meta-analysis of 7 randomized clinical trials among 4013 patients with advanced-stage epithelial ovarian cancer responding to first-line platinum-based chemotherapy, progression-free survival improved in most subgroups but not in homologous recombination-proficient tumors, and no subgroup demonstrated a statistically significant overall survival benefit. Efficacy and toxic effects varied across agents.

**Meaning:**

This study found that first-line PARP inhibitor maintenance therapy was associated with a progression-free survival benefit in many patient populations; however, the lack of overall survival benefit, increased toxic effects, and variability across subgroups and regimens, suggest that treatment should be individualized.

## Teaser

This systematic review and meta-analysis investigates the association of first-line poly(adenosine diphosphate-ribose) polymerase inhibitor (PARP inhibitor) maintenance therapy with efficacy and safety outcomes among patients with advanced-stage epithelial ovarian cancer.

## Introduction

Advanced epithelial ovarian, tubal, or peritoneal cancer (EOC) remains one of the most lethal malignant tumors, with little improvement in the 5-year survival rate over the past 3 decades.^1,2,3,4,5^ Current standard of care includes primary cytoreductive surgery (PCS) followed by platinum-based chemotherapy (with or without bevacizumab) or neoadjuvant chemotherapy (NACT) followed by interval cytoreductive surgery and further chemotherapy (with or without bevacizumab).^6^ These approaches, while effective in achieving initial disease control, often fail to prevent recurrence in most patients.

The introduction of poly(adenosine diphosphate-ribose) polymerase inhibitors (PARP inhibitors) has transformed the treatment landscape and management of EOC, particularly for patients with high-grade serous *BRCA*-variant and homologous recombination-deficient (HRD) advanced-stage EOC by targeting DNA repair.^7,8,9,10,11,12,13,14,15^ PARP inhibitors are now established as standard maintenance therapy after first-line treatment and in recurrent platinum-sensitive EOC.^6,16^

However, the long-term benefits and risks associated with PARP inhibitor remain uncertain. While a 2021 Cochrane review with meta-analysis by Tattersall et al^17^ indicated that PARP inhibitor maintenance therapy may be associated with improved PFS in newly diagnosed and recurrent platinum-sensitive EOC, the association with overall survival (OS) was uncertain due to immature data. However, as OS data from key randomized clinical trials (RCTs) have matured,^12,18,19^ more comprehensive insights are emerging. Analysis of these updated findings, along with new RCTs evaluating the use of PARP inhibitors in newly diagnosed EOC with no evidence of disease or a response to first-line platinum-based chemotherapy^20,21,22^ is critical to evaluate the long-term outcomes associated with PARP inhibitor maintenance and refine treatment strategies.

In addition, it remains unclear to what extent PARP inhibitors provide benefit across clinical and molecular subgroups and patients undergoing different treatment approaches, such as PCS vs NACT. As alternative therapeutic approaches, such as antibody drug conjugates,^23,24^ emerge, understanding the relative efficacy and safety of PARP inhibitor in the broader context of treatment of patients with EOC is crucial given that there are subsets of patients who clearly require more effective treatment options beyond PARP inhibitor maintenance.

This systematic review and meta-analysis evaluated the efficacy and safety of first-line PARP inhibitor maintenance therapy in advanced-stage EOC, assessing survival outcomes (PFS and OS) and high-grade adverse events. It also explored subgroup-specific outcomes by *BRCA* and HRD status, surgical approach (PCS vs NACT), response to platinum-based chemotherapy, residual disease status, and specific PARP inhibitor type used.

## Methods

### Overview

We conducted a systematic review and meta-analysis to evaluate the efficacy and safety of PARP inhibitor maintenance therapy for advanced-stage EOC with no evidence of disease or a response to first-line platinum-based chemotherapy. Institutional review board approval was not required for this study given that it used publicly available data sources and did not involve individual-level patient data. All included studies adhered to the ethical standards of their respective institutional or national research committees and complied with the 1964 Helsinki Declaration and its subsequent amendments. Informed consent was obtained from participants in the original studies included in this meta-analysis, as documented in the respective publications. The reporting of the review adhered to the Preferred Reporting Items for Systematic Reviews and Meta-Analyses (PRISMA) reporting guideline. A protocol detailing study objectives, inclusion criteria, and analytical approach was registered with the International Prospective Register of Systematic Reviews (PROSPERO; CRD42024627450).

### Search Strategy

An information specialist (C.A.-H.) composed search strategies for Embase (embase.com), Medline (Ovid), the Web of Science Core Collection (webofscience.com), the Cochrane Central Register of Controlled Trials (Cochrane library), and clinicaltrials.gov followed by peer review by a second information specialist (eAppendix in Supplement 1). Search syntax was translated from Embase-Elsevier by publicly available macros.^25^ No restrictions on language or publication date were applied. Searches were conducted on August 20, 2024; references were exported to EndNote 21 (Clarivate Analytics) and deduplicated using Deduklick.^26^ Titles and abstracts were screened independently by 2 reviewers (S.P. and T.A.Z.), with full-text assessment and conflict resolution as described in the eMethods in Supplement 1.

### Eligibility and Inclusion Criteria

We included studies that enrolled patients with advanced-stage EOC who had no evidence of disease or had achieved complete or partial response to first-line platinum-based chemotherapy. Studies were eligible if they examined the use of PARP inhibitors as maintenance therapy compared with placebo or no maintenance therapy after standard chemotherapy (SC) with or without bevacizumab.

Eligible study designs included RCTs and prospective 2-armed observational studies. To be included, studies had to report at least 1 of the primary outcomes, namely PFS or OS. Studies were restricted to those involving adult patients (aged ≥18 years).

### Outcome Measures

Primary outcomes were PFS and OS. We defined PFS as the time from randomization to disease progression or death, whichever occurred first, and OS as the time interval between randomization and death from any cause or the date of the last follow-up. Outcomes were compared between interventions using hazard ratios (HRs) with 95% CIs, which express the relative risk (RR) of an event occurring between groups. Incidence of any event (death or recurrence) and incidence of death were also compared. Secondary outcomes included the incidence of severe adverse events, specifically those with grades higher than 3, as defined by the Common Terminology Criteria for Adverse Events (CTCAE).

### Statistical Analysis

#### Data Extraction

Data were extracted independently by 2 reviewers (S.P. and T.A.Z.). Any disagreements were resolved through discussion or, if necessary, consultation with a third reviewer (M.F.) to reach a consensus.

#### Risk of Bias and Quality of Evidence

The methodological quality of included individual studies was assessed using the risk of bias in prospective randomized studies (RoB) tool^27^ (eMethods in Supplement 1). The overall quality of the evidence for primary and secondary outcomes was assessed using the Grading of Recommendations, Assessment, Development, and Evaluations (GRADE) framework^28,29^ (eMethods in Supplement 1).

#### Summary Measures and Synthesis of Results

For each outcome, the number of events in each group and corresponding summary statistics (RR or HR and 95% CIs) were extracted or calculated. In cases where summary statistics were not provided, RRs were derived from the number of events and participants in each group. When the total number of events was not directly reported, it was estimated using the available summary statistic and CI. Data synthesis was conducted using a random-effects model with the restricted maximum likelihood estimator to account for study variability.^30^ CIs were calculated using the Hartung-Knapp-Sidik-Jonkman method.^31^ All statistical tests were 2-sided, and a *P* value < .05 was considered statistically significant. Heterogeneity across studies was assessed using the τ^2^, inconsistency index-squared (*I*^2^), and 95% prediction intervals (PIs), which estimate the expected range of true effect sizes in future studies.^32,33^ All statistical analyses and visualizations were performed using R statistical software version 4.1.3 (R Project for Statistical Computing).

#### Subgroup Analyses

Primary and secondary outcomes were analyzed for patients with HRD tumors, *BRCA*-variant tumors, *BRCA*–wild type tumors, and homologous recombination proficient (HRP) tumors. Further subgroups were based on the extent of residual disease (complete vs incomplete resection), response to first-line treatment (complete vs partial response), and timing of first-line chemotherapy (NACT vs PCS).

#### Publication Bias

To address potential reporting biases, such as publication bias, we planned a priori to use funnel plots and the Egger test^34,35^ if at least 10 studies were included in the meta-analysis. However, given that 7 RCTs were included in this analysis, this assessment of publication bias was not conducted.

#### Descriptive Comparative Analysis of PARP Inhibitors

Separate pairwise meta-analysis was performed to compare descriptively the relative effectiveness and safety profiles of different PARP inhibitor regimens compared with SC. This was done by evaluating the incidence of any event, death, and high-grade adverse events in the overall population and HRD, *BRCA-*variant, *BRCA*–wild type, and HRP subgroups (eMethods in Supplement 1).

## Results

### Study Selection

**Figure 1.**
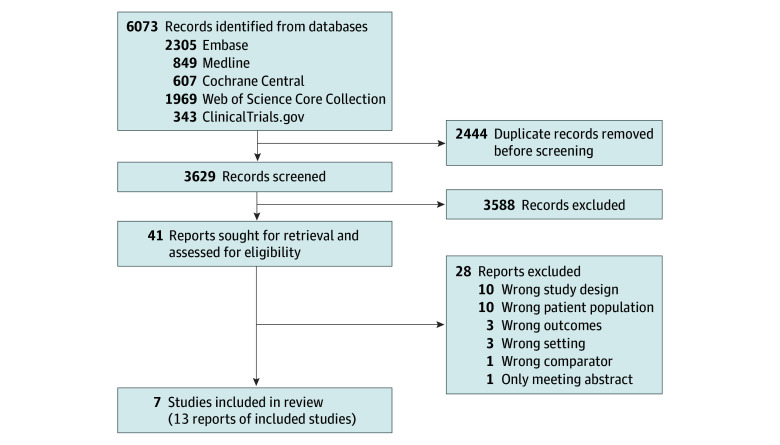
Study Selection Flowchart This flowchart depicts the study inclusion process. A total of 6073 records were identified from databases and hand searches. After removal of duplicates and screening for relevance, 41 full-text articles were assessed, and 7 studies reported in 13 publications were included in the final analysis.

Figure 1 presents the flowchart of study selection. The search strategy (eAppendix in Supplement 1) retrieved 3629 unique titles. After we screened titles and abstracts, 3588 studies were excluded for irrelevance. We assessed 41 full-text articles, with 28 studies excluded due various reasons, mostly incorrect study design and incorrect patient population (Figure 1). Ultimately, 13 reports from 7 RCTs involving 4013 patients (2619 patients receiving PARP inhibitor maintenance and 1394 patients receiving SC alone) met inclusion criteria.^11,12,13,15,18,19,20,21,22,36,37,38,39^

### Study Characteristics, Risk of Bias, and Individual Study Results

All included trials were judged to have a low RoB (eTable 1 in Supplement 1). Their characteristics are summarized in eTable 2 in Supplement 1.

#### Synthesis of Results

##### Overall Population

**Figure 2.**
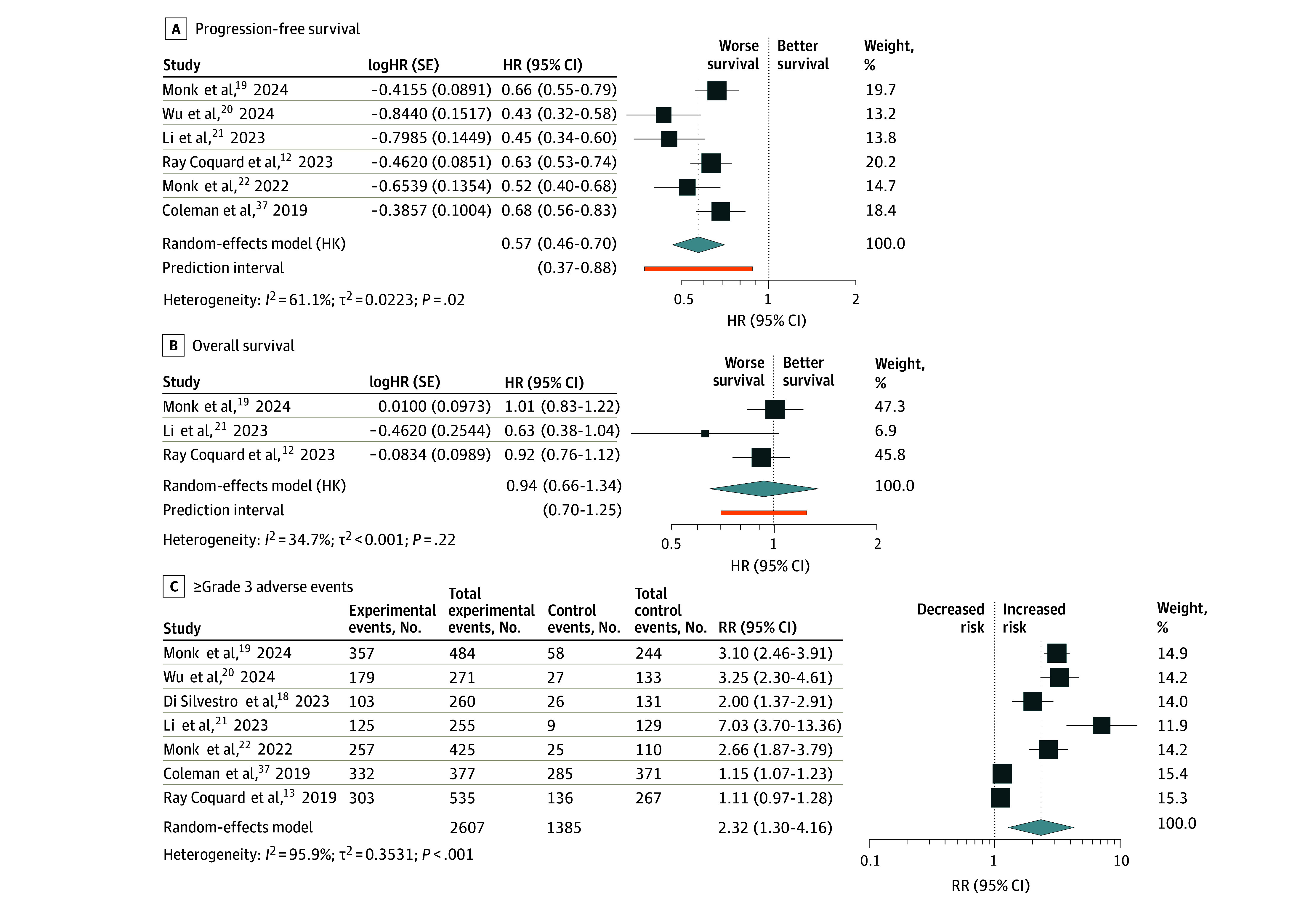
Outcomes in the Overall Population The forest plot shows hazard ratios (HRs), 95% CIs, and 95% prediction intervals for progression-free survival (A) and overall survival (B) and risk ratios (RRs) for grade 3 or higher adverse events in the overall population (C). HK indicates Hartung-Knapp; SE, standard error.

PARP inhibitor maintenance with SC was associated with improved PFS (HR, 0.57; 95% CI, 0.46-0.70; 6 studies^12,19,20,22,37,40^ and 3622 patients) and a reduced rate of recurrence or death (RR, 0.76; 95% CI, 0.64-0.89; 6 studies^12,19,20,22,37,40^ and 3622 patients) compared with SC alone (Table; Figure 2A; eFigure 1 in Supplement 1). However, OS was not significantly different among the overall population (HR, 0.94; 95% CI, 0.66-1.34; 3 studies^12,19,21^ and 1923 patients), while mortality rates were comparable (RR, 0.93; 95% CI, 0.77-1.12; 4 studies^12,19,20,21^ and 2314 patients) (Table; Figure 2B; eFigure 1 in Supplement 1). Severe adverse events (≥3) were significantly more frequent in the PARP inhibitor group (RR, 2.40; 95% CI, 1.16-4.93; 7 studies^13,18,19,20,21,22,37^ and 3601 patients) (Figure 2C; Table).

**Table.** Summary of Findings and Certainty of Evidence

Outcome	Anticipated absolute effect size, %		Relative effect size (95% CI)	*I*^2^ (95% CI), %^a^	τ^2^ (95% CI)^a^	Participants, No. (studies, No.)	Certainty of evidence (GRADE)^b^
	Risk associated with PARP inhibitor maintenance	Risk associated with only SC					
PFS hazard risk (overall)	NA	NA	0.57 (0.46-0.70)	61.1 (5.0-84.1)	0.02 (0.00-0.23)	3622 (6 studies) ^12,19,20,22,37,40^	High
OS hazard risk (overall)	NA	NA	0.94 (0.66-1.34)	34.7 (0.0-78.8)	0.00 (0.00-2.42)	1923 (3 studies) ^12,19,21^	Moderate^c^
Recurrence or death (overall)	56.1	72.7	0.76 (0.64-0.89)	73.5 (39.3-88.4)	0.02 (0.00-0.17)	3622 (6 studies) ^12,19,20,22,37,40^	High
Death (overall)	41.8	47.6	0.93 (0.77-1.12)	30.6 (0.0-74.9)	0.00 (0.00-0.43)	2314 (4 studies) ^12,19,20,21^	Moderate^c^
Grade 3-4 adverse event (overall)	66.2	43.5	2.40 (1.16-4.93)	96.5 (94.3-97.8)	0.43 (0.01-2.90)	3601 (7 studies) ^13,18,19,20,21,22,37^	High
PFS hazard risk (HRD)	NA	NA	0.44 (0.39-0.50)	0.0 (0.0-74.6)	0.00 (0.00-0.62)	1833 (6 studies) ^12,19,20,21,22,37^	High
OS hazard risk (HRD)	NA	NA	0.79 (0.43-1.45)	46.1 (0.0-84.1)	0.04 (0.00-2.01)	1017 (3 studies) ^12,19,21^	Moderate^c^
Recurrence or death (HRD)	45.5	67.4	0.68 (0.61-0.77)	31.6 (0.0-72.3)	0.00 (0.00-0.20)	1833 (6 studies) ^12,19,20,21,22,37^	High
Death (HRD)	34.8	42.3	0.82 (0.53-1.28)	44.4 (0.0-83.4)	0.02 (0.00-1.00)	1017 (3 studies) ^12,19,21^	Moderate^c^
PFS hazard risk (*BRCA* variant)	NA	NA	0.40 (0.35-0.45)	44.4 (0.0-70.8)	0.00 (0.00-0.03)	1429 (7 studies) ^11,12,19,20,21,22,37^	High
OS hazard risk (*BRCA* variant)	NA	NA	0.67 (0.33-1.37)	54.4 (0.0-86.9)	0.05 (0.00-3.23)	851 (3 studies) ^12,18,19^	High
Recurrence or death (*BRCA* variant)	35.5	56.6	0.64 (0.59-0.70)	0.0 (0.0-70.8)	0.00 (0.00-0.02)	1429 (7 studies) ^11,12,19,20,21,22,37^	High
Death (*BRCA* variant)	35.7	48.6	0.74 (0.44-1.26)	52.9 (0.0-86.5)	0.02 (0.00-1.76)	851 (3 studies) ^12,18,19^	High
Grade 3-4 adverse event (*BRCA* variant)	57.4	37.8	1.49 (0.06-37.78)	83.7	0.01	601 (2 studies)^11,36^	Low^c^^,^^d^
PFS hazard risk (*BRCA* wild type)	NA	NA	0.62 (0.44-0.86)	67.5 (15.8-87.4)	0.05 (0.00-0.56)	1596 (5 studies) ^13,19,20,21,37^	High
OS hazard risk (*BRCA* wild type)	NA	NA	1.12 (0.33-3.74)	0.0	0.00	426 (2 studies)^12,19^	Low^c^^,^^d^
Recurrence or death (*BRCA* wild type)	53.6	69.7	0.76 (0.63-0.91)	65.1 (16.0-85.5)	0.02 (0.00-0.22)	2165 (6 studies) ^13,19,20,21,22,37^	High
Death (*BRCA* wild type)	66.1	62.9	1.04 (0.57-1.89)	0.0	0.00	426 (2 studies)^12,19^	Low^c^^,^^d^
PFS hazard risk (HRP)	NA	NA	0.74 (0.51-1.06)	62.2 (0.0-85.7)	0.04 (0.00-0.84)	1098 (5 studies) ^13,15,21,22,37^	High
OS hazard risk (HRP)	NA	NA	1.05 (0.22-5.25)	20.5	0.01	526 (2 studies)^12,19^	Low^c^^,^^d^
Recurrence or death (HRP)	56.9	70.1	0.92 (0.85-0.99)	0.0 (0.0-79.2)	0.00 (0.00-0.03)	982 (5 studies) ^13,15,21,22,37^	Moderate^c^
Death (HRP)	74.5	72.1	1.03 (0.92-1.15)	0.0	0.00	526 (2 studies)^12,19^	Low^c^^,^^d^
PFS hazard risk (complete response)	NA	NA	0.50 (0.39-0.66)	40.5 (0.0-78.0)	0.03 (0.00-0.26)	1423 (5 studies) ^13,20,21,22,38^	High
Recurrence or death (complete response)	49.8	70.0	0.69 (0.54-0.88)	71.6 (28.2-88.8)	0.03 (0.00-0.33)	1423 (5 studies) ^13,20,21,22,38^	High
PFS hazard risk (partial response)	NA	NA	0.57 (0.37-0.89)	53.9 (0.0-83.0)	0.07 (0.00-0.87)	655 (5 studies) ^13,20,21,22,38^	High
Recurrence or death (partial response)	71.6	82.0	0.89 (0.80-0.99)	0.0 (0.0-79.2)	0.00 (0.00-0.14)	655 (5 studies) ^13,20,21,22,38^	High
PFS hazard risk (NACT)	NA	NA	0.51 (0.31-0.84)	66.0 (0.4-88.4)	0.06 (0.00-1.37)	1213 (4 studies) ^13,15,20,21^	High
Recurrence or death (NACT)	49.6	68.9	0.72 (0.64-0.81)	0.0 (0.0-84.7)	0.00 (0.00-0.08)	1213 (4 studies) ^13,15,20,21^	High
PFS hazard risk (PCS)	NA	NA	0.54 (0.36-0.81)	41.8 (0.0-80.4)	0.02 (0.00-1.11)	1055 (4 studies) ^13,15,20,21^	High
Recurrence or death (PCS)	41.2	62.4	0.65 (0.39-1.08)	76.3 (35.0-91.4)	0.08 (0.01-1.56)	1055 (4 studies) ^13,15,20,21^	High
PFS hazard risk (complete cytoreduction)	NA	NA	0.51 (0.44-0.60)	62.2 (0.0-79.2)	0.04 (0.00-0.11)	1500 (5 studies) ^13,20,21,22,39^	High
Recurrence or death (complete cytoreduction)	43.7	64.8	0.68 (0.60-0.77)	0.0 (0.0-79.2)	0.00 (0.00-0.09)	1500 (5 studies) ^13,20,21,22,39^	High
PFS hazard risk (incomplete cytoreduction)	NA	NA	0.39 (0.24-0.62)	65.5 (9.8-86.8)	0.09 (0.00-1.01)	904 (5 studies)^13,20,21,22,39^	High
Recurrence or death (incomplete cytoreduction)	51.1	76.5	0.63 (0.43-0.93)	79.8 (52.2-91.4)	0.07 (0.01-0.88)	904 (5 studies) ^13,20,21,22,39^	High

Abbreviations: GRADE, Grading of Recommendations Assessment, Development, and Evaluation; HRD, homologous recombination deficient; HRP, homologous recombination proficient; NA, not applicable; NACT, neoadjuvant chemotherapy; OS, overall survival; PARP inhibitor, poly(adenosine diphosphate-ribose) polymerase inhibitor; PCS, primary cytoreductive surgery; PFS, progression-free survival; RR, relative risk; SC, standard chemotherapy.

^a^ I^2^ and τ^2^ confidence intervals are reported only when more than 2 studies were included in the analysis.

^b^ GRADE categories were defined as high (we are very confident that the true effect size lies close to that of the estimate of the effect size), moderate (we are moderately confident in the effect size estimate; the true effect size is likely to be close to the estimate of the effect size, but there is a possibility that it is substantially different), low (our confidence in the effect size estimate is limited; the true effect size may be substantially different from the estimate of the effect size), and very low (we have very little confidence in the effect size estimate; the true effect size is likely to be substantially different from the estimate of effect size) certainty.

^c^ The 95% CI for the pooled effect sizes include the unit.

^d^ Very small number of events.

##### HRD Subgroup

**Figure 3.**
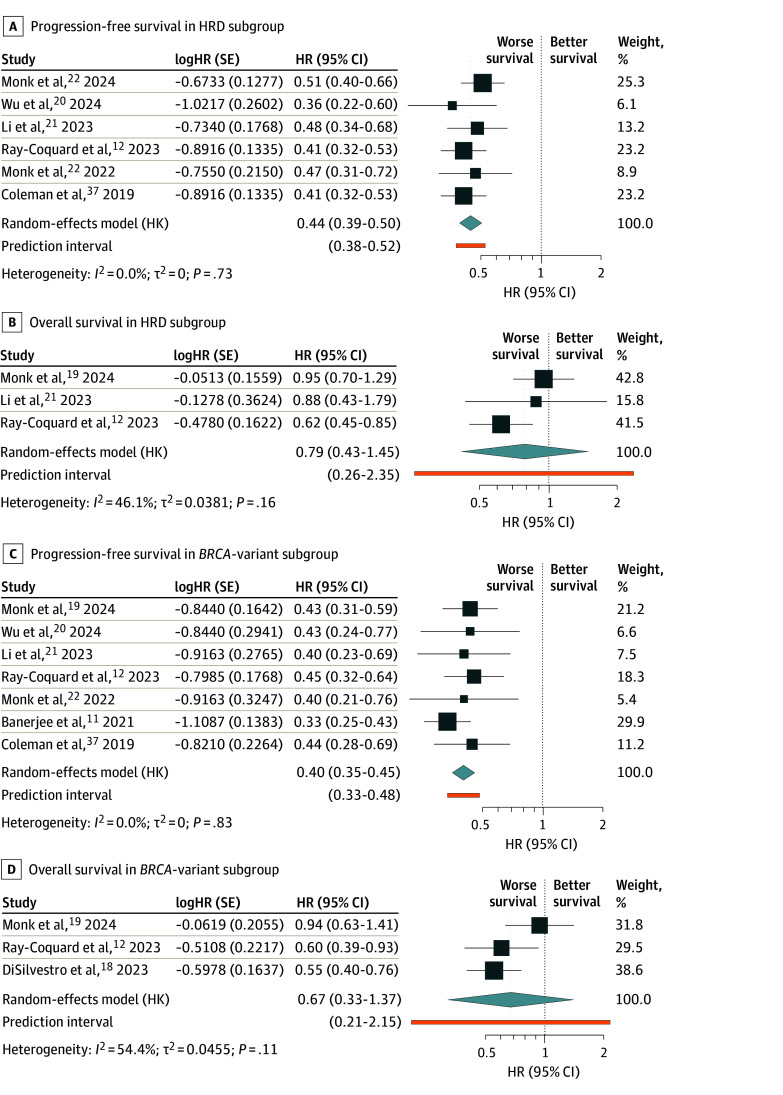
Outcomes in Homologous Recombination-Deficient (HRD) and *BRCA*-Variant Subgroups The forest plot shows hazard ratios (HRs), 95% CIs, and 95% prediction intervals for progression-free survival (A) and overall survival (B) in the HRD population and for progression-free survival (C) and overall survival (D) in the *BRCA*-variant population. HK indicates Hartung-Knapp; SE, standard error.

Results in the HRD subgroup were consistent with those of the overall population. PARP inhibitor maintenance with SC was associated with improved PFS (HR, 0.44; 95% CI, 0.39-0.50; 6 studies^12,19,20,21,22,37^ and 1833 patients) and a reduced recurrence rate (RR, 0.68; 95% CI, 0.60-0.77; 6 studies^12,19,20,21,22,37^ and 1833 patients) compared with SC alone (Table; Figure 3A; eFigure 2 in Supplement 1). However, OS showed no statistically significant improvement (HR, 0.79; 95% CI, 0.43-1.45, 3 studies,^12,19,21^ 1017 patients), nor did mortality (RR, 0.82; 95% CI, 0.53-1.28; 3 studies^12,19,21^ and 1017 patients) (Table; Figure 3B; eFigure 2 in Supplement 1). There were insufficient data to assess adverse events in this subgroup.

##### *BRCA*-Variant Subgroup

In this subgroup, PARP inhibitor maintenance was associated with improved PFS (HR, 0.40; 95% CI, 0.35-0.45; 7 studies^11,12,19,20,21,22,37^ and 1429 patients), decreased rates of any event (RR, 0.64; 95% CI, 0.59-0.70; 7 studies^11,12,19,20,21,22,37^ and 1429 patients) compared with SC alone, but was not associated with improved OS (HR, 0.67; 95% CI, 0.33-1.37; 3 studies^12,18,19^ and 851 patients) (Table; Figure 3C; eFigure 3 in Supplement 1). Mortality was also not statistically significantly reduced (RR, 0.74; 95% CI, 0.44-1.26; 3 studies^12,18,19^ and 851 patients) (Table, Figure 3D; eFigure 3 in Supplement 1). There was no statistically significant increase in severe adverse events in the PARP inhibitor group (RR, 1.49; 95% CI, 0.06-37.78; 2 studies^11,36^ and 601 patients) (Table; eFigure 3 in Supplement 1).

##### *BRCA*–Wild Type Subgroup

In this subgroup, PARP inhibitor maintenance was associated with improved PFS (HR, 0.62; 95% CI, 0.44-0.86; 5 studies^13,19,20,21,37^ and 1596 patients) compared with SC alone, although the PI included the null (95% PI, 0.31-1.23). PARP inhibitor maintenance was also associated with a reduced rate of any event (RR, 0.76; 95% CI, 0.63-0.91; 6 studies^13,19,20,21,22,37^ and 2165 patients) (Table; eFigure 4 in Supplement 1). However, there was no association with OS (HR, 1.12; 95% CI, 0.33-3.74; 2 studies^12,19^ and 426 patients), nor was there with mortality (RR, 1.04; 95% CI, 0.57-1.89; 2 studies^12,19^ and 426 patients) (Table; eFigure 4 in Supplement 1). There were insufficient data to assess adverse events in this subgroup.

##### HRP Subgroup

PARP inhibitor maintenance was not associated with improved PFS in this subgroup (HR, 0.74; 95% CI, 0.51-1.06; 5 studies^13,15,21,22,37^ and 1098 patients), but it was associated with a reduced rate of any event (RR: 0.92, 95%CI: 0.86-0.99, 5 studies,^13,15,21,22,37^ 1098 patients) compared with SC alone (Table; eFigure 5 in Supplement 1). There was no significant difference in the rate of death or OS between the 2 study groups in this population (Table; eFigure 5 in Supplement 1).

#### Subgroup and Sensitivity Analyses

##### Complete and Partial Response

PARP inhibitor maintenance was associated with improved PFS in patients with a complete response to chemotherapy (HR, 0.50, 95% CI:0.39-0.66, 5 studies,^13,20,21,22,38^ 1423 patients) and patients with a partial response to chemotherapy (HR, 0.57; 95% CI, 0.37-0.89; 5 studies^13,20,21,22,38^ and 655 patients), with a reduced rate of any event (Table; eFigure 6 in Supplement 1). However, the PI for the partial response group included the null (95% PI, 0.24-1.39) (eFigure 6 in Supplement 1). There were insufficient data to perform analyses for OS, rate of death, or adverse events.

##### NACT and PCS

PARP inhibitor maintenance was associated with improved PFS and a reduced rate of any event compared with SC alone in patients with NACT (HR, 0.51; 95% CI, 0.36-0.81; RR, 0.72; 95% CI, 0.64-0.81; 4 studies^13,15,20,21^ and 1213 patients) (Table; eFigure 7 in Supplement 1). However, the PI for PFS included the null (95% PI, 0.20-1.30) (eFigure 7 in Supplement 1). In patients with PCS, PARP inhibitor maintenance was associated with improved PFS but not a reduction in the rate of any event (HR, 0.54; 95% CI, 0.36-0.81; RR, 0.65; 95% CI, 0.39-1.08; 4 studies^13,15,20,21^ and 1055 patients) (Table; eFigure 7 in Supplement 1). There were insufficient data to perform analyses for OS, rate of death, or adverse events.

##### Optimal and Suboptimal Cytoreduction

PARP inhibitor maintenance was associated with improved PFS in patients with optimal cytoreduction (HR, 0.51, 95%CI: 0.44-0.60, 5 studies,^13,20,21,22,39^ 1500 patients) and suboptimal cytoreduction (HR, 0.39; 95% CI, 0.25-0.62; 5 studies^13,20,21,22,39^ and 904 patients), with a reduced rate of any event (Table; eFigure 8 in Supplement 1). However, the PI for the suboptimal cytoreduction group included the null (95% PI, 0.15-1.03) (eFigure 8 in Supplement 1). There were insufficient data to perform analyses for OS, rate of death, or adverse events.

#### Overall Quality of Evidence and Publication Bias

The quality of the evidence was high for PFS and any event rate for all examined populations except the HRP subgroup, where incidence of any event was characterized by moderate quality (Table). Quality of evidence was high for any grade 3 or higher event for the overall population but low for the *BRCA*-variant subgroup (Table). Quality of evidence for OS and overall death was high for the *BRCA*-variant subgroup but moderate for the overall and HRD population and low for the *BRCA*–wild type subgroup (Table).

#### Descriptive Comparative Analysis of PARP Inhibitors

No discrepancies in study or participant characteristics or the definition of interventions or outcomes were found (eTable 2 in Supplement 1). Network geometry showed that all PARP inhibitor regimens were compared with SC alone, with no direct comparison between different PARP inhibitor regimens (eFigure 9 in Supplement 1).

**Figure 4.**
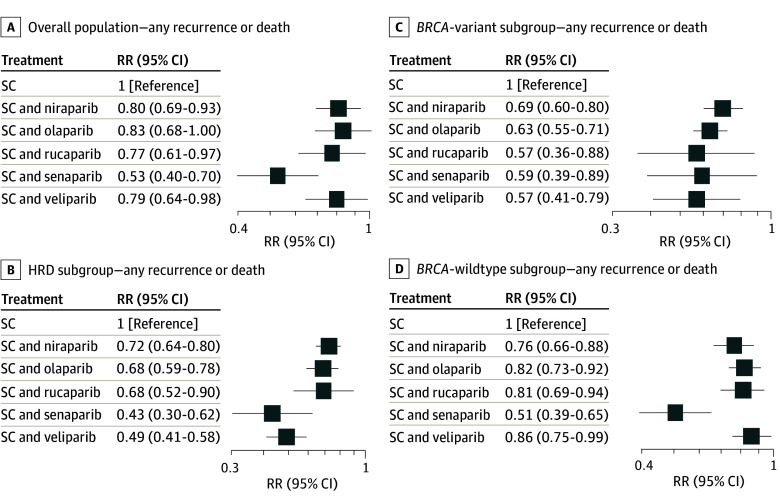
Comparative Analysis of Study Drugs Comparative analysis of poly(adenosine diphosphate-ribose) polymerase inhibitors (PARP inhibitors) for the risk of recurrence or death across different PARP inhibitor regimens in the overall population (A), homologous recombination-deficient (HRD) subgroup (B), *BRCA*-variant subgroup (C), and *BRCA*-wildtype subgroup (D). RR indicates relative risk; SC, standard chemotherapy.

In the overall population, all PARP inhibitor maintenance regimens were more effective in regards to any event than SC alone. Senaparib demonstrated the lowest observed RR (0.53; 95% CI, 0.40-0.70), followed by rucaparib (0.77; 95% CI, 0.61-0.97), while olaparib had the highest RR (0.83; 95% CI, 0.68-1.00) (Figure 4A). Similarly, in the HRD subgroup, all PARP inhibitor maintenance regimens were more effective than SC alone, with senaparib again showing the lowest RR, followed by olaparib (Figure 4B). Among patients with *BRCA*-variant tumors, all PARP inhibitor maintenance regimens were more effective than SC alone, while veliparib showed the lowest observed RR, followed by rucaparib (Figure 4C). For the *BRCA-*wildtype subgroup, all PARP inhibitor maintenance regimens were more effective than SC alone, with senaparib showing the lowest observed RR and niraparib in second place (Figure 4D).

Regarding incidence of death, senaparib in the overall population and olaparib in HRD and *BRCA*-variant subgroups were associated with the lowest observed mortality risk (eFigure 10 in Supplement 1). For the incidence of high-grade adverse events, olaparib (RR, 1.34; 95% CI, 0.87-2.08) and veliparib (RR, 1.15; 95% CI, 0.64-2.06) showed the lowest risk and niraparib showed the highest risk (RR, 4.73; 95% CI, 2.77-8.07) among all PARP inhibitor regimens in their respective comparisons (eFigure 10 in Supplement 1).

## Discussion

This systematic review and network meta-analysis provides several major insights. First, we found that first-line PARP inhibitor maintenance was associated with improved PFS in most molecular subgroups in advanced-stage EOC, with the exception of HRP tumors, and no subgroups demonstrated a statistically significant improvement in OS. Second, PARP inhibitor maintenance was associated with increased high-grade adverse events in any category of patients. Third, observed differences in treatment effect estimates indicated that certain patient subgroups may benefit more from specific PARP inhibitor regimens, underscoring the need for individualized treatment approaches. For example, olaparib and veliparib were identified as the regimens with the lowest toxic effects compared with other PARP inhibitors in their respective studies, providing a potentially safer option in clinical practice.

Consistent PFS benefits observed across diverse clinical scenarios, such as surgical approach (PCS vs NACT), chemotherapy response, or residual disease status after surgical cytoreduction, demonstrated the broad applicability of PARP inhibitors. However, the lack of statistically significant PFS improvement for HRP tumors, along with the PI not crossing the null in the *BRCA*-wildtype subgroup, suggest that the benefit may not be uniform across populations. These findings, combined with the increased incidence of high-grade adverse events with PARP inhibitor maintenance, warrant careful evaluation of the balance between survival benefits and potential toxic effects.^41^ Notably, data on high-grade adverse events remained limited in some key subgroups, such as HRD and HRP populations. Additionally, quality of life outcomes, which are critical for maintenance therapies given their long duration, are underreported.

The lack of statistically significant OS benefits in any molecular subgroup, including patients with *BRCA*-variant and HRD tumors, may reflect the association of subsequent therapies with survival after progression.^42,43^ Most RCTs have been powered for PFS with limited long-term follow up to assess OS outcomes comprehensively. In our analysis, OS data were available for 4 of 7 included trials. Ideally, future studies should prioritize reporting mature OS data to fully capture the association of maintenance therapies with median OS and event-free survival at various landmarks, such as 5, 7, and 10 years.

These findings advocate for a paradigm shift from a one-PARP inhibitor-fits-all approach to a right PARP inhibitor for the right patient strategy. Although formal statistical comparisons between PARP inhibitor regimens were not performed due to a lack of direct comparison, observed variation in efficacy and safety differences among PARP inhibitor regimens suggest that individual agents may be better suited to certain patient populations. While senaparib demonstrated a lower estimated risk of progression compared with SC in patients with HRD tumors, olaparib was a safer option in these patients, raising the clinical question of whether efficacy or safety should take precedence. However, such differences should be interpreted cautiously. Notably, the use of bevacizumab in the PAOLA-1 trial^13^ introduces variability given that its synergistic associations with PARP inhibitors may influence efficacy and toxic effects, complicating comparisons across studies. Furthermore, the FLAMES trial,^20^ which investigated maintenance therapy with senaparib, was relatively small (404 patients) and was carried out in a uniformly ethnic Chinese population of patients, raising questions about broader applicability.

Varying efficacy and safety profiles of different PARP inhibitor regimens in different clinical and molecular populations, coupled with the increased incidence of adverse events, emphasize the importance of personalized treatment strategies.^44,45^ Future research should prioritize refining patient stratification through advanced biomarker identification and assess quality-adjusted survival as the primary outcome for maintenance therapies.^46,47,48,49^

### Limitations

This study has several limitations. First, no direct comparisons between different PARP inhibitor regimens were available, and a network meta-analysis was not feasible. Second, OS data were limited to 4 of 7 trials, and although PIs for OS did not cross the null, wide CIs around *I*^2^ and τ^2^ indicate uncertainty in long-term-survival estimates. Third, adverse events and quality-of-life reporting were inconsistent across subgroups, limiting a comprehensive assessment of tolerability and patient-centered outcomes associated with PARP inhibitor maintenance therapy. Fourth, RRs were derived from cumulative event data at differing follow-up times, limiting comparability across trials and introducing uncertainty in pooled estimates.

## Conclusions

In this meta-analysis of 7 RCTs, no molecular subgroup demonstrated a statistically significant OS benefit with first-line PARP inhibitor maintenance therapy in EOC, and findings suggest that the consistency of PFS benefit may vary, particularly among patients with *BRCA*-wildtype or HRP tumors. The observed variability in efficacy, toxic effects, and long-term outcomes across subgroups and PARP inhibitor regimens suggests that treatment decisions should be individualized. The identification of predictive biomarkers and inclusion of patient-centered outcomes, such as quality-adjusted survival, should be prioritized to guide optimal maintenance strategies.

## Notes

Supplement 1. **eFigure 1.** Outcomes in the Overall Population **eFigure 2.** Outcomes in the HRD Subgroup **eFigure 3.** Outcomes in the *BRCA*-Variant Subgroup **eFigure 4.** Outcomes in the *BRCA*–Wild Type Subgroup **eFigure 5.** Outcomes in the HRP Subgroup **eFigure 6.** Outcomes by Chemotherapy Response Status **eFigure 7.** Outcomes by Primary Treatment Status **eFigure 8.** Outcomes by Cytoreduction Status **eFigure 9.** Geometry of the Network **eFigure 10.** Comparative Analysis of PARP inhibitors **eTable 1.** Assessment of Risk of Bias Across Domains Using the Cochrane Collaboration Risk of Bias (RoB-2). **eTable 2.** PICOS Characteristics of Included Studies
eMethods.
**eAppendix.** Search Strategies

Supplement 2.
Data Sharing Statement
