# Supplementary material for: PARP Inhibitor Maintenance After First-Line Chemotherapy in Advanced-Stage Epithelial Ovarian Cancer: A Systematic Review and Meta-Analysis
Source: JAMA Netw Open. 2025 Nov 5;8(11):e2541648. doi: 10.1001/jamanetworkopen.2025.41648 (PMC12590296; doi:10.1001/jamanetworkopen.2025.41648)
Supplement: Supplement 1. — eFigure 1. Outcomes in the Overall Population eFigure 2. Outcomes in the HRD Subgroup eFigure 3. Outcomes in the BRCA-Variant Subgroup eFigure 4. Outcomes in the BRCA–Wild Type Subgroup eFigure 5. Outcomes in the HRP Subgroup eFigure 6. Outcomes by Chemotherapy Response Status eFigure 7. Outcomes by Primary Treatment Status eFigure 8. Outcomes by Cytoreduction Status eFigure 9. Geometry of the Network eFigure 10. Comparative Analysis of PARP inhibitors eTable 1. Assessment of Risk of Bias Across Domains Using the Cochrane Collaboration Risk of Bias (RoB-2). eTable 2. PICOS Characteristics of Included Studies eMethods. eAppendix. Search Strategies [file jamanetwopen-e2541648-s001.pdf]

## SUPPLEMENTARY ONLINE CONTENT

Petousis S, Kahramanoglu I, Appenzeller-Herzog C, et al. PARP Inhibitor maintenance after first-line chemotherapy in advanced-stage epithelial ovarian cancer: a systematic review and meta-analysis. *JAMA Netw Open*. 2025;8(11):e2541648. doi:10.1001/jamanetworkopen.2025.41648

eFigure 1. Outcomes in the Overall Population
eFigure 2. Outcomes in the HRD Subgroup
eFigure 3. Outcomes in the BRCA-Variant Subgroup
eFigure 4. Outcomes in the BRCA–Wild Type Subgroup
eFigure 5. Outcomes in the HRP Subgroup
eFigure 6. Outcomes by Chemotherapy Response Status
eFigure 7. Outcomes by Primary Treatment Status
eFigure 8. Outcomes by Cytoreduction Status
eFigure 9. Geometry of the Network
eFigure 10. Comparative Analysis of PARP inhibitors
eTable 1. Assessment of Risk of Bias Across Domains Using the Cochrane Collaboration Risk of Bias (RoB-2).
eTable 2. PICOS Characteristics of Included Studies
eMethods.
eAppendix. Search Strategies
eReferences.

**This supplemental material has been provided by the authors to give readers additional information about their work.**

© 2025 Petousis S et al. *JAMA Network Open*.

**eFigure 1 Outcomes in the Overall Population** Forest plot showing risk ratios (RR) and 95% confidence interval (CI) for **A.** any event and **B.** any death in the overall population.

**A.**

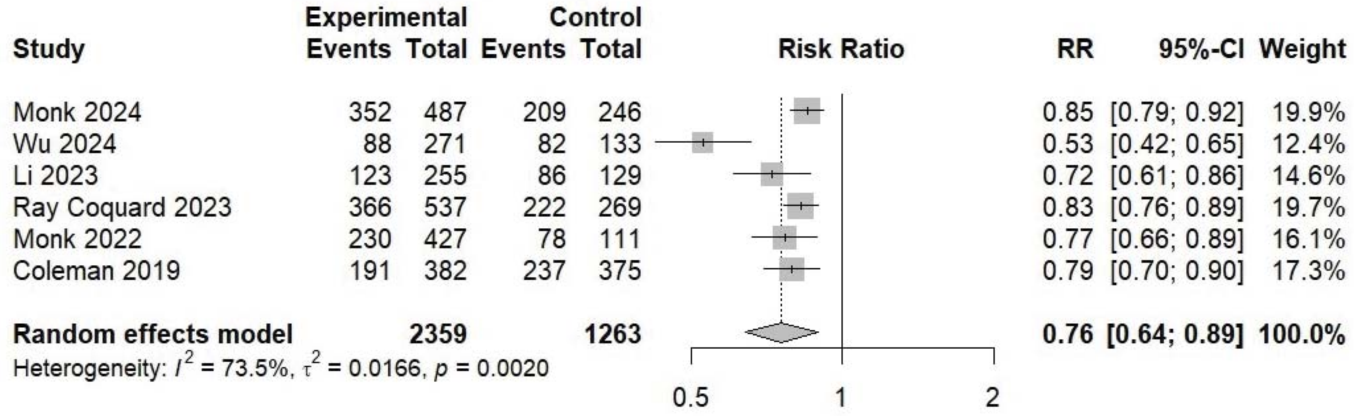


**B.**

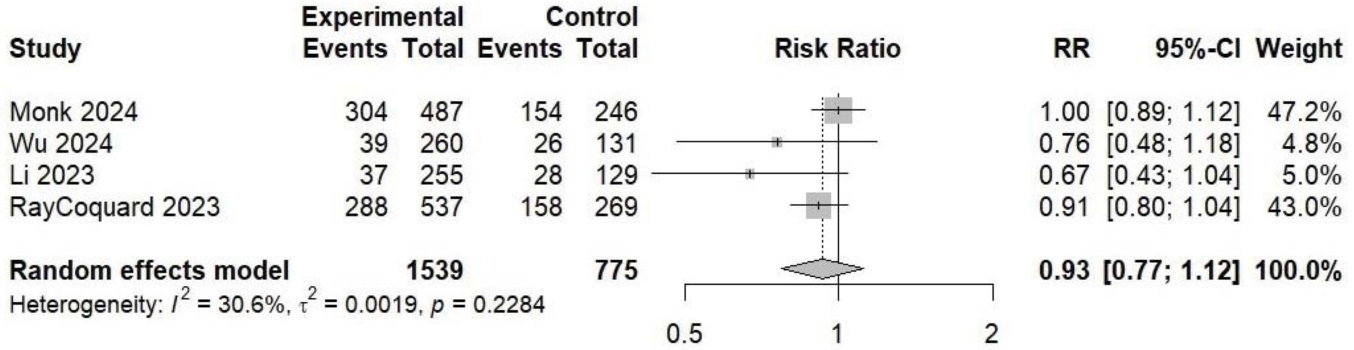


© 2025 Petousis S et al. *JAMA Network Open*.

**eFigure 2 Outcomes in the HRD Subgroup** Forest plot of risk ratios (RR) and 95% confidence interval (CI) for **A.** progression-free survival and **B.** overall survival in the *HRD* population.

**A.**

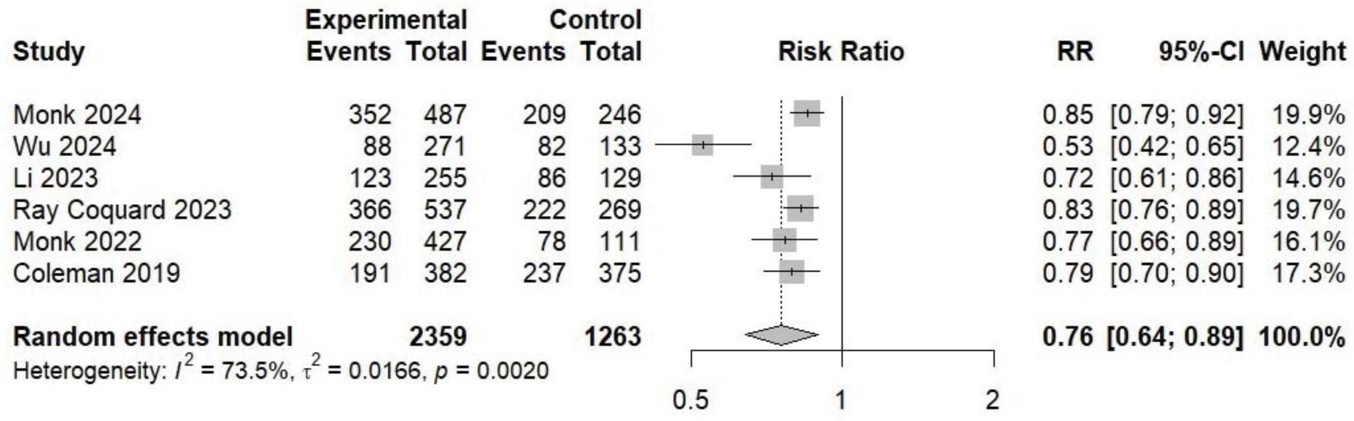


**B.**

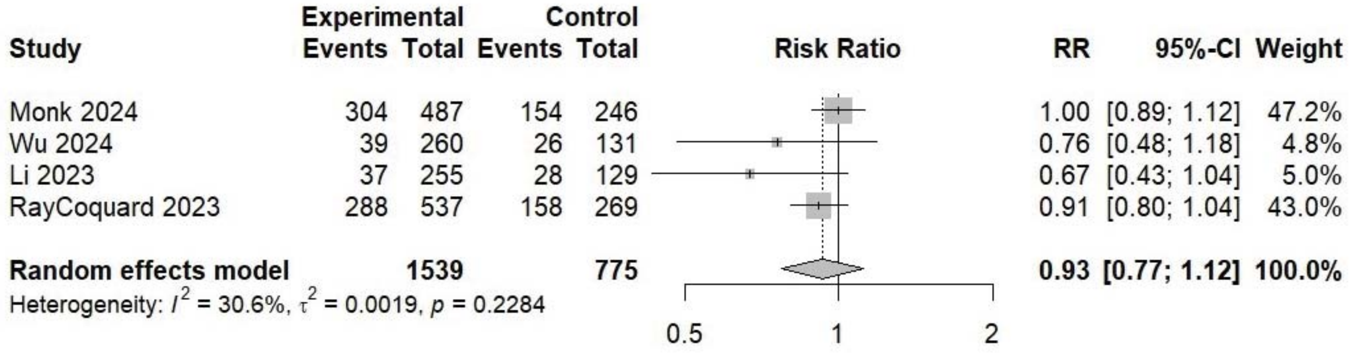


© 2025 Petousis S et al. *JAMA Network Open*.

**eFigure 3 Outcomes in the *BRCA*-variant Subgroup** Forest plot of risk ratios (RR) and 95% confidence interval (CI) for **A.** any event, **B.** any death, and **C.** for grade 3 or higher adverse events in the *BRCA*-mutated population.

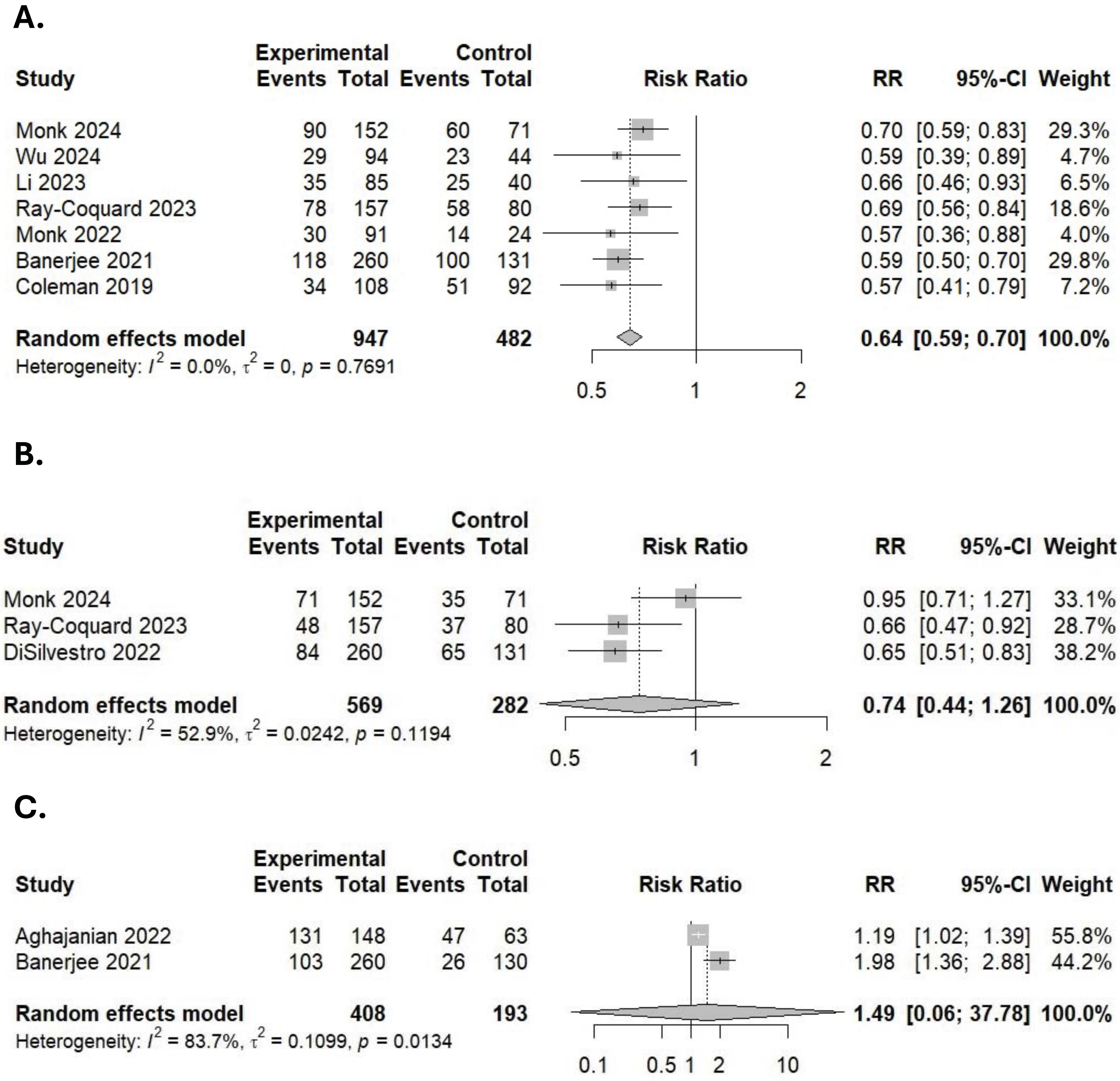


© 2025 Petousis S et al. *JAMA Network Open*.

**eFigure 4 Outcomes in the *BRCA*-wild type Subgroup** Forest plot of hazard ratios (HR), 95% confidence interval (CI), and prediction interval for **A.** progression-free survival, **B.** overall survival. Forest plot of risk ratios (RR) and CI for **C.** any event, **D.** any death, in the *BRCA*-wildtype population.

## A.

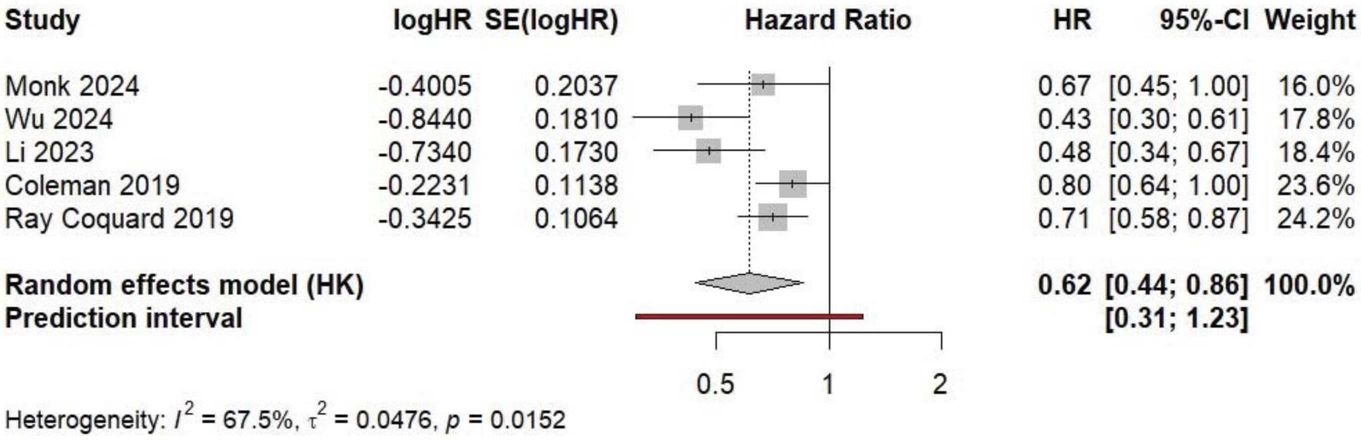


## B.

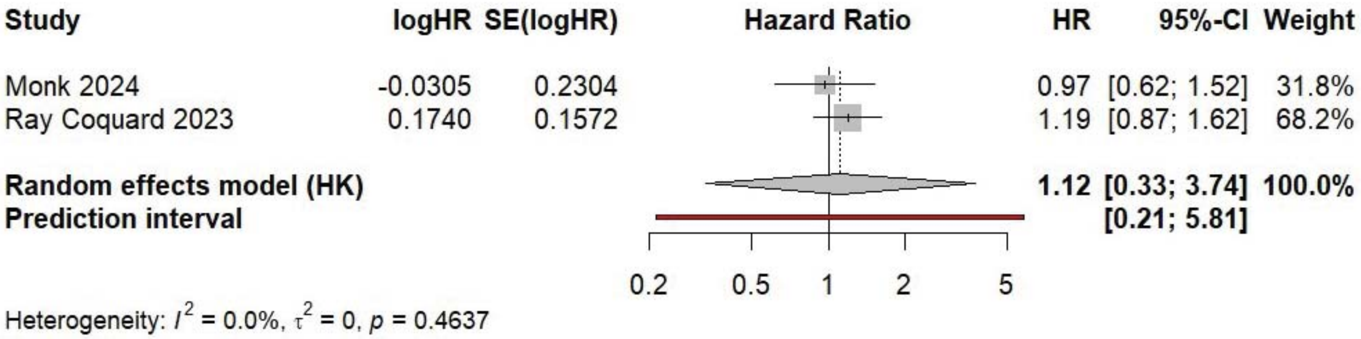


## C.

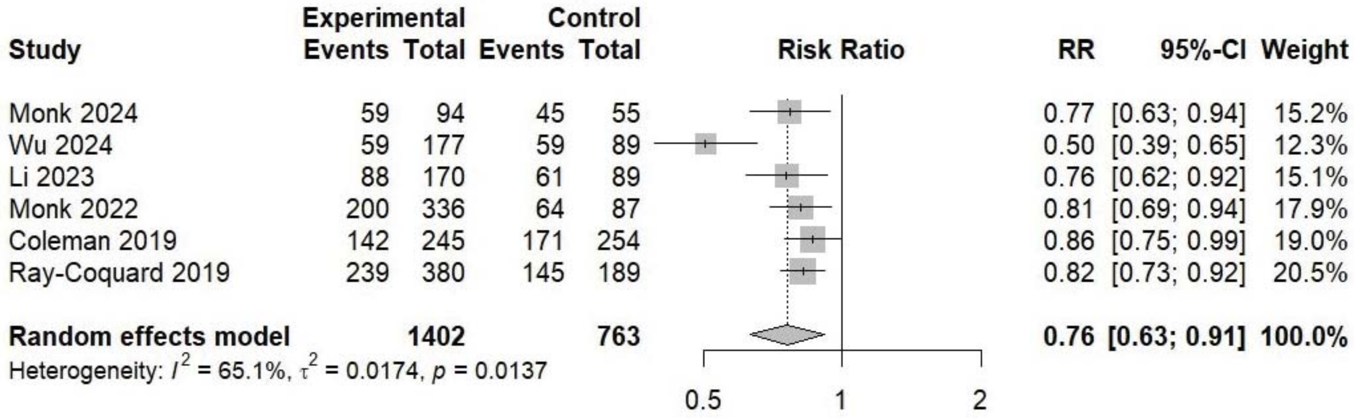


## D.

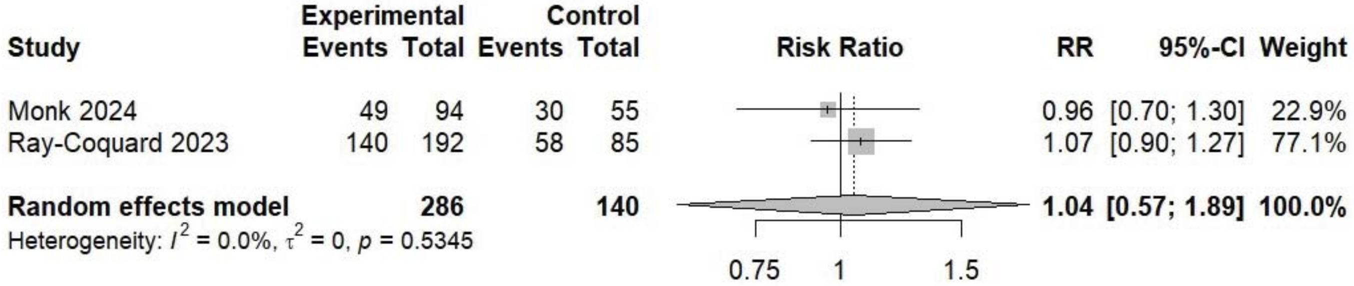


© 2025 Petousis S et al. *JAMA Network Open*.

**eFigure 5 Outcomes in the HRP Subgroup** Forest plot of hazard ratios (HR), 95% confidence interval (CI), and prediction interval for **A.** progression-free survival, **B.** overall survival. Forest plot of risk ratios (RR) and CI for **C.** any event, **D.** any death, in the HRP population.

**A.**

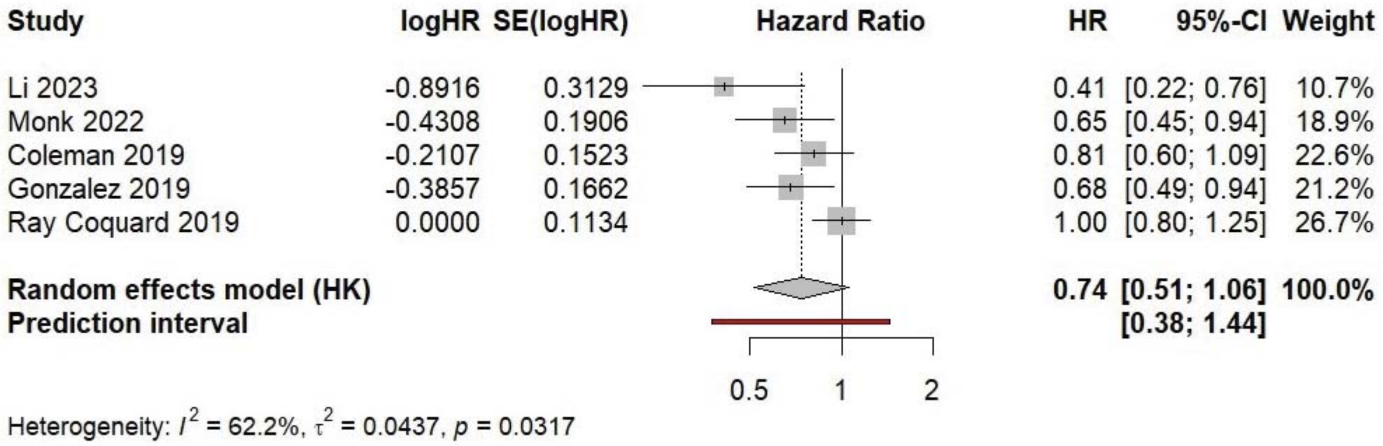


**B.**

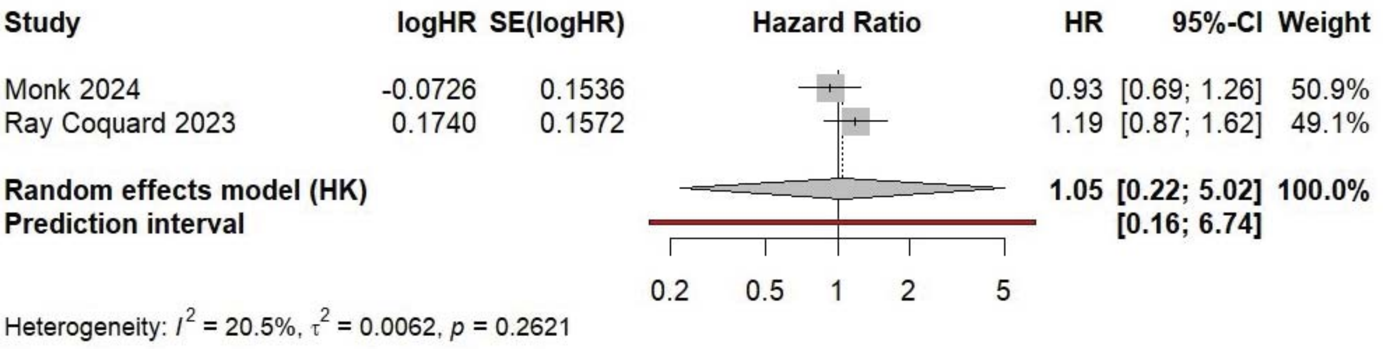


**C.**

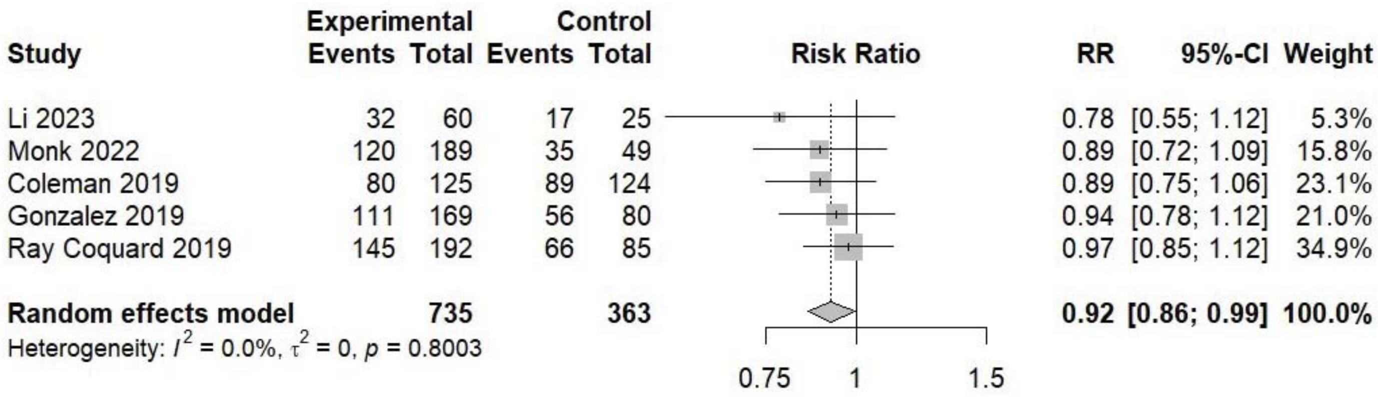


**D.**

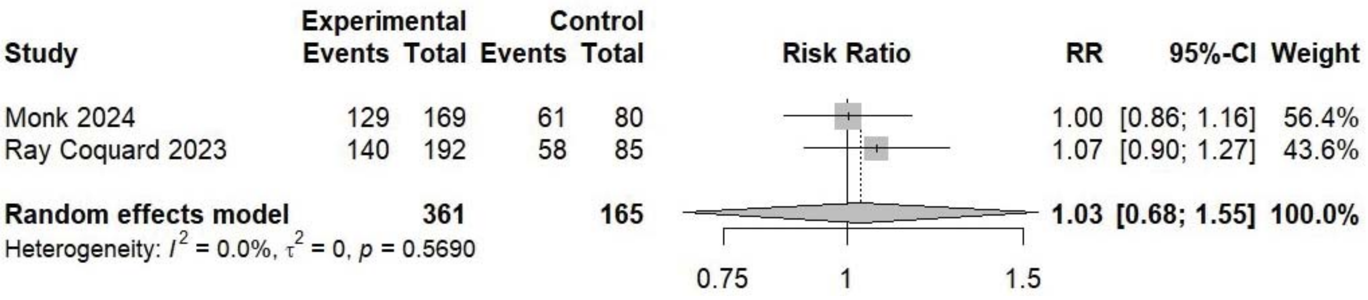


© 2025 Petousis S et al. *JAMA Network Open*.

**eFigure 6 Outcomes according to Chemotherapy Response Status** Forest plot of hazard ratios (HR), 95% confidence interval (CI), and prediction interval for progression-free survival for **A.** complete and **B.** partial response. Forest plot of risk ratios (RR) and CI for any event for **C.** complete and **D.** partial response.

**A.**

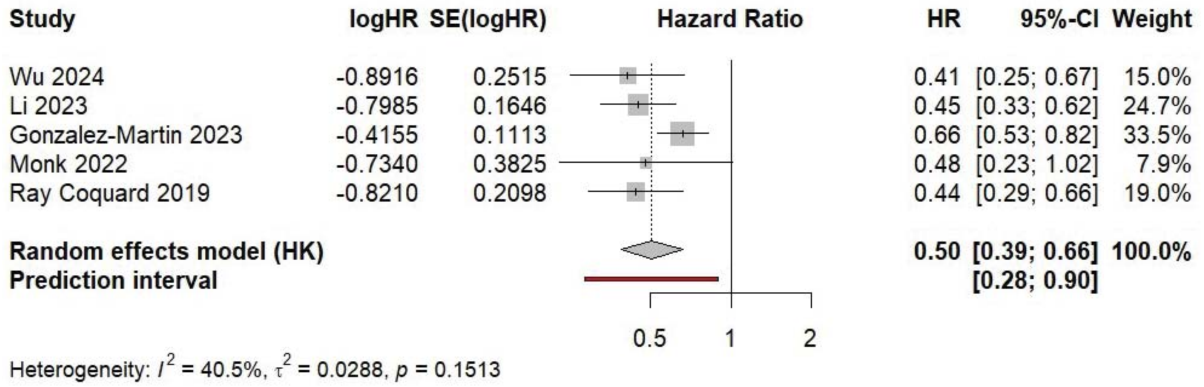


**B.**

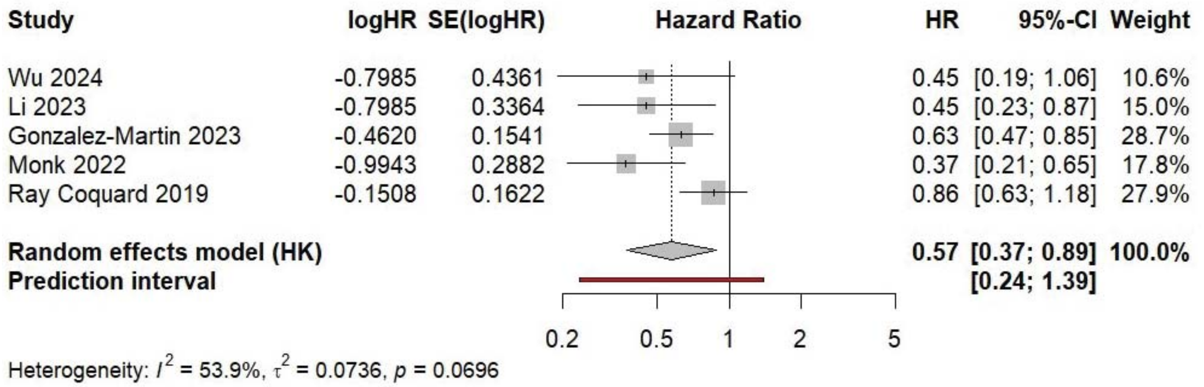


**C.**

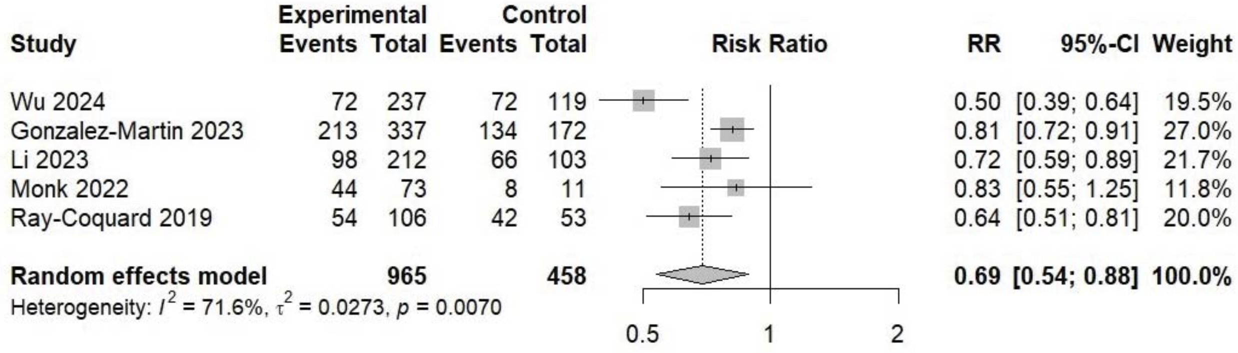


**D.**

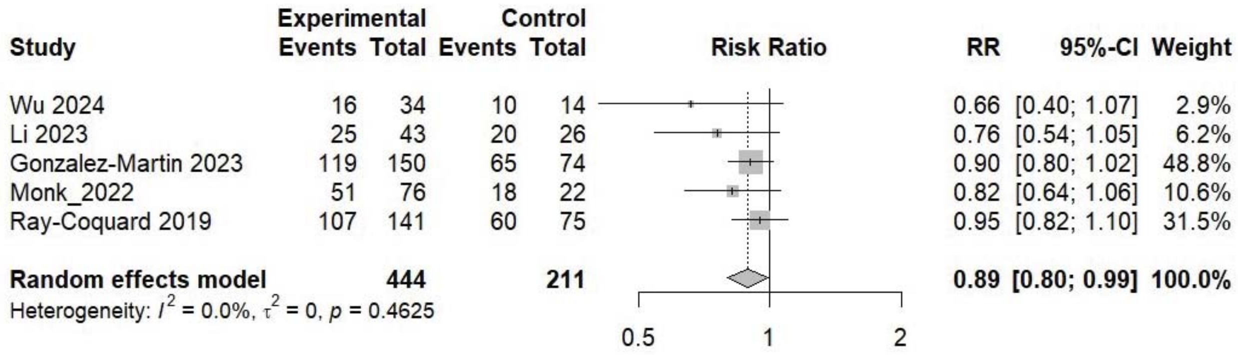


© 2025 Petousis S et al. *JAMA Network Open*.

**eFigure 7 Outcomes by Primary Treatment Status** Forest plot of hazard ratios (HR), 95% confidence interval (CI), and prediction interval for progression-free survival for **A.** primary cytoreductive surgery and **B.** neoadjuvant chemotherapy. Forest plot of risk ratios (RR) and CI for any event for **C.** primary cytoreductive surgery and **D.** neoadjuvant chemotherapy.

**A.**

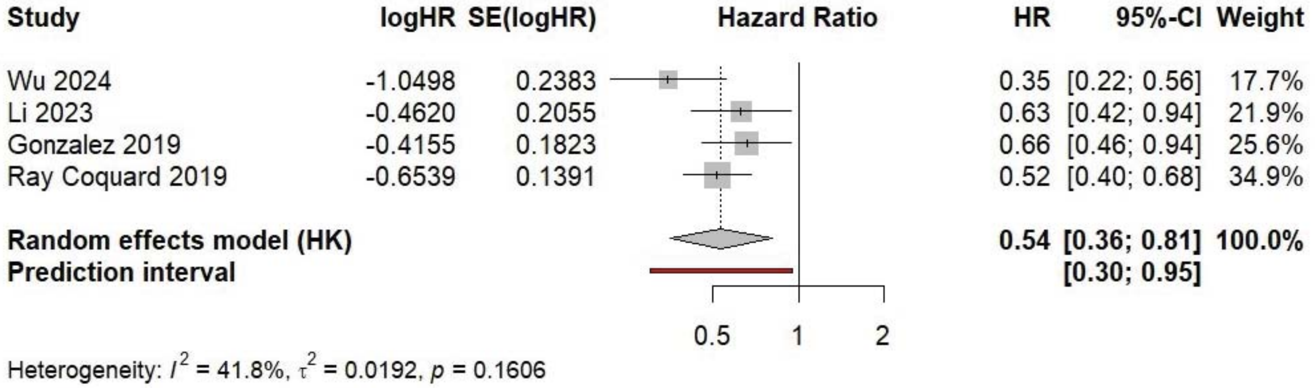


**B.**

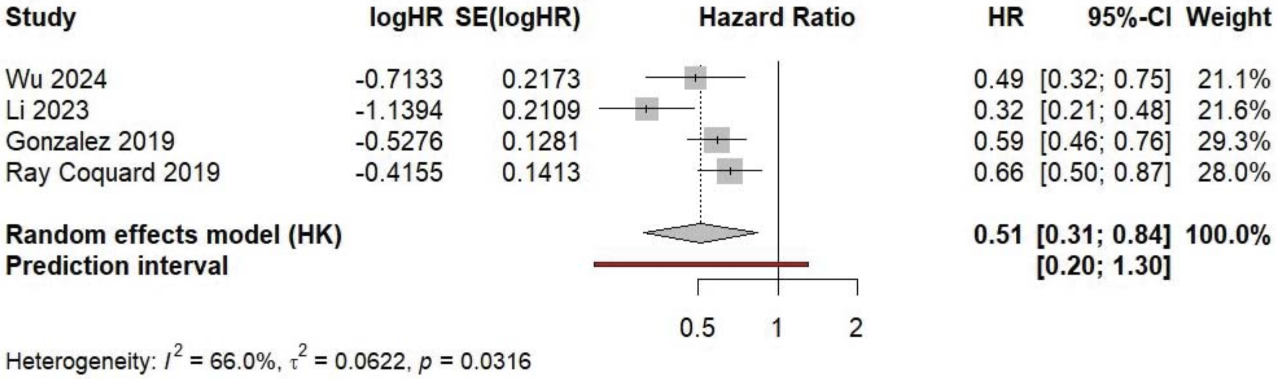


**C.**

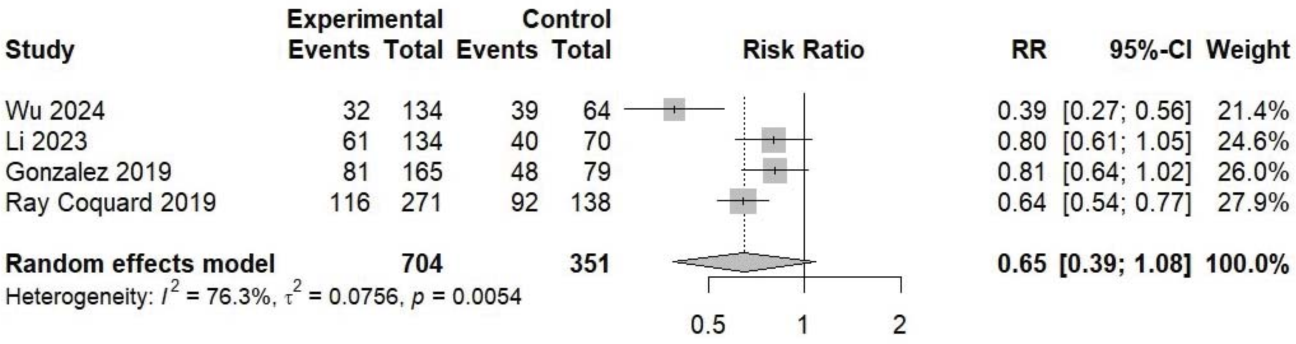


**D.**

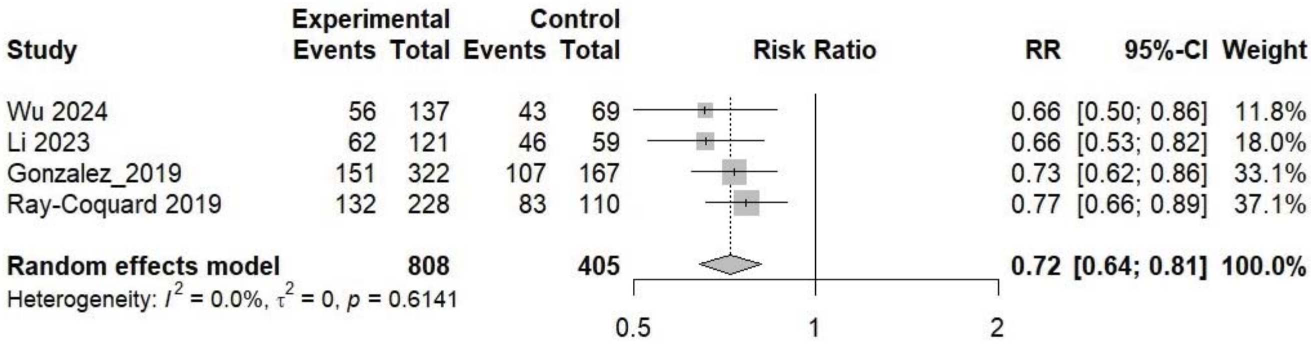


© 2025 Petousis S et al. *JAMA Network Open*.

**eFigure 8 Outcomes by Cytoreduction Status** Forest plot of hazard ratios (HR), 95% confidence interval (CI), and prediction interval for progression-free survival for **A.** optimal cytoreduction and **B.** suboptimal cytoreduction. Forest plot of risk ratios (RR) and CI for any event for **C.** optimal cytoreduction and **D.** suboptimal cytoreduction.

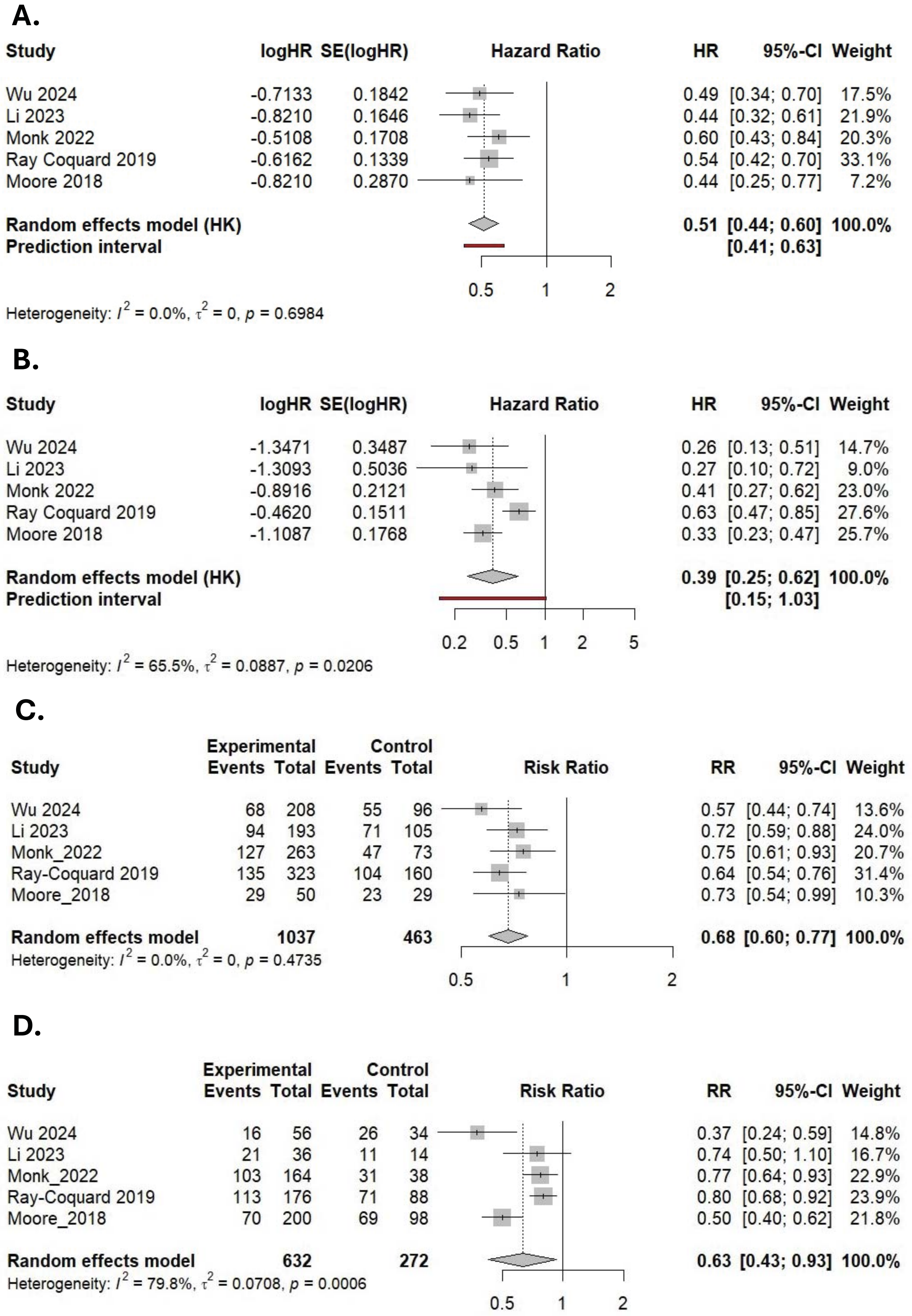


© 2025 Petousis S et al. *JAMA Network Open*.

**eFigure 9 Geometry of the network** Geometry of the indirect comparison showing pairs of comparisons between PARP inhibitor maintenance regimens and standard chemotherapy alone.
*SC=Standard chemotherapy*

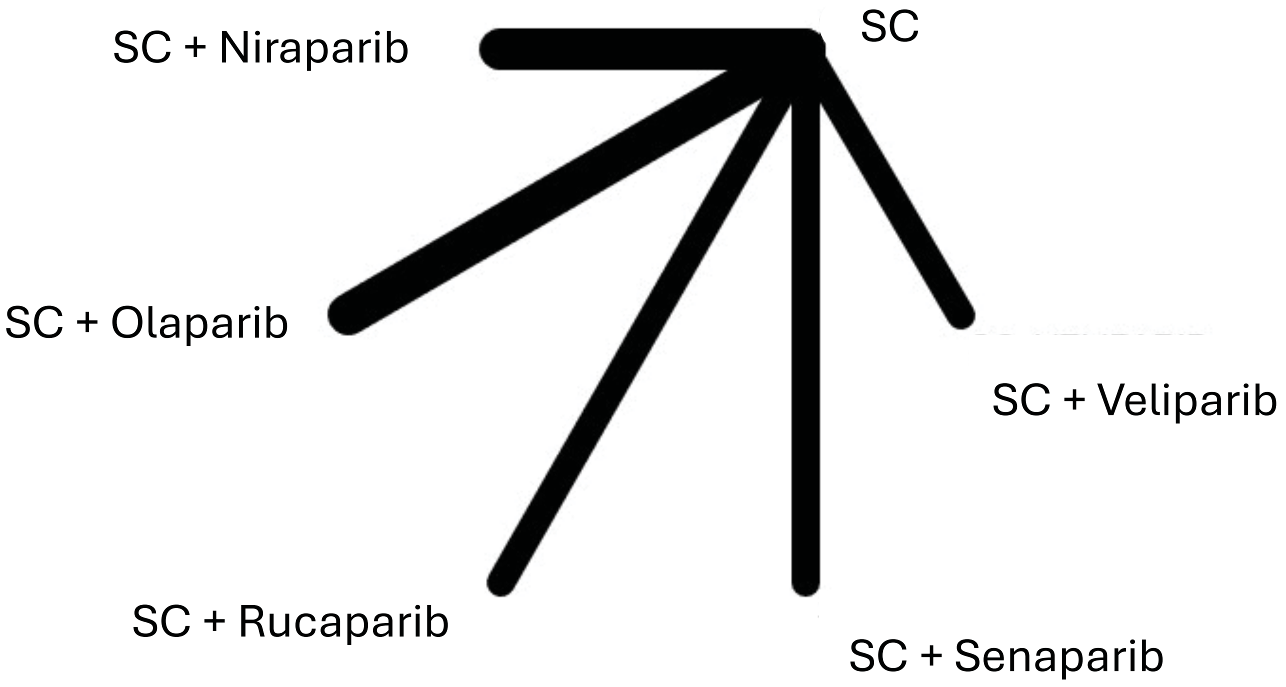


© 2025 Petousis S et al. *JAMA Network Open*.

**eFigure 10 Comparative Analysis of PARP inhibitors** Comparative analysis of PARP inhibitors for the risk of any death across different PARP inhibitor regimens in the **A.** overall population, **B.** HRD subgroup, and **C.** *BRCA*-mutated subgroup and **D.** the risk for grade 3 or higher adverse events across different PARP inhibitor regimens in the overall population.
*SC=Standard chemotherapy, CI=Confidence Interval, RR=Relative risk, HRD=Homologous recombination deficient*

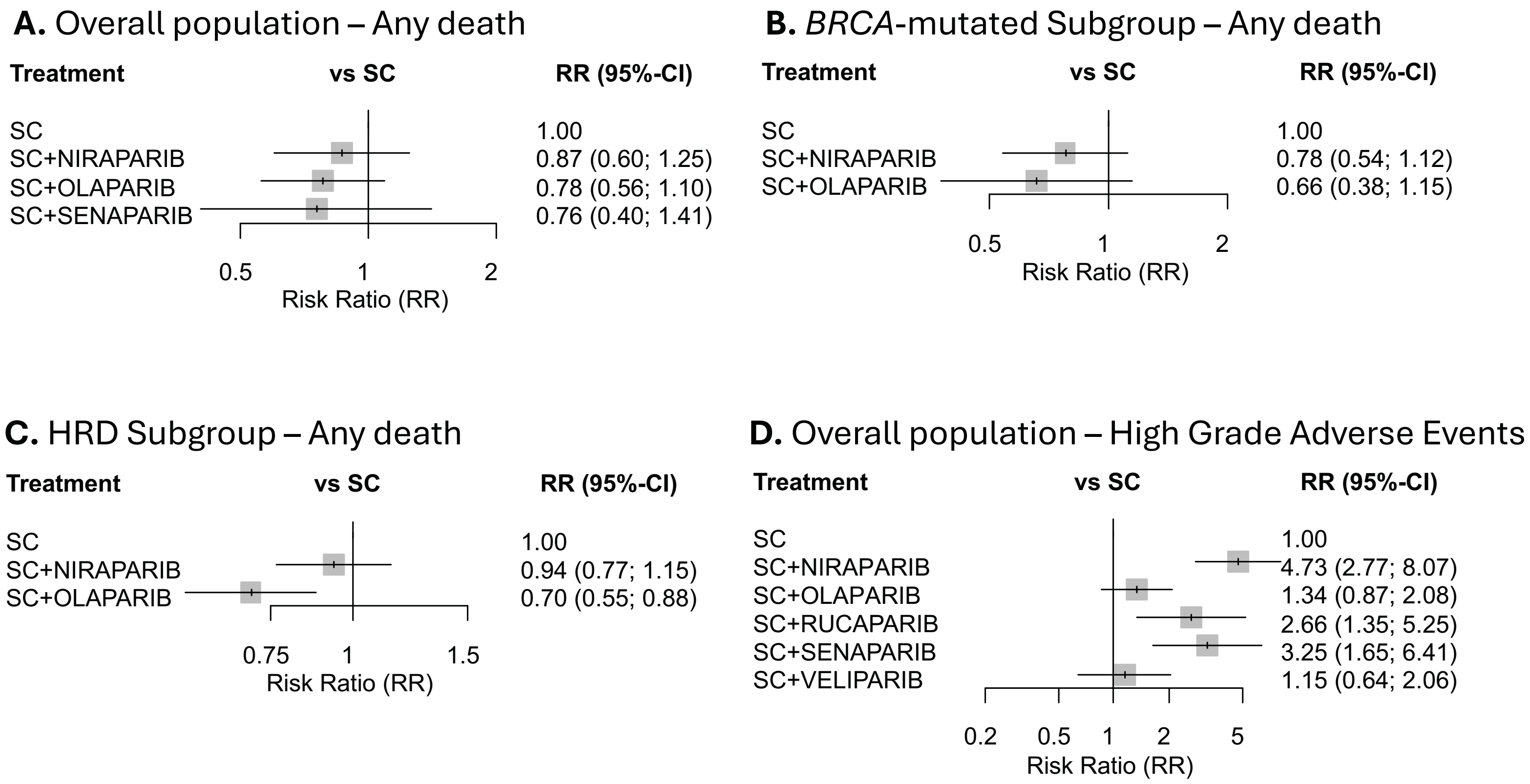


© 2025 Petousis S et al. *JAMA Network Open*.

**eTable 1.** Assessment of risk of bias across domains using the Cochrane Collaboration Risk of Bias (RoB-2)

| **Bias Domain** | **Signalling Questions** | Coleman et al,[37] 2019 | Ray-Coquard et al,[13] 2019 | Banerjee et al,[11] 2021 | Aghajanian et al,[36] 2022 | Di Silvestro et al,[18] 2023 | Monk et al,[22] 2022 | Gonzalez-Martin et al,[38] 2023 | Li et al,[21] 2023 | Ray-Coquard et al,[12] 2023 | Wu et al,[20] 2024 | Monk et al,[19] 2024 | Gonzalez-Martin et al,[15] 2019 | Moore et al,[39] 2018 |
|---|---|---|---|---|---|---|---|---|---|---|---|---|---|---|
| **Domain 1a: Risk of bias arising from the randomization process** | 1a.1 Was the allocation sequence random? | Y | Y | Y | Y | Y | Y | Y | Y | Y | Y | Y | Y | Y |
| | 1a.2 Was the allocation sequence concealed until patients were enrolled and assigned to interventions? | Y | Y | Y | Y | Y | Y | Y | Y | Y | Y | Y | Y | Y |
| | 1a.3 Did baseline differences between intervention groups suggest a problem with the randomization process? | N | N | N | N | N | N | N | N | N | N | N | N | N |
| **Domain 1b: Risk of bias arising from the timing of identification or recruitment of participants in a cluster-** | 1b.1 Were all the individual participants identified and recruited (if appropriate) before randomization? | Y | Y | Y | Y | Y | Y | Y | Y | Y | Y | Y | Y | Y |
| | | | | | | | | | | | | | | |

© 2025 Petousis S et al. *JAMA Network Open*.

| **Bias Domain** | **Signalling Questions** | Coleman et al,[37] 2019 | Ray-Coquard et al,[13] 2019 | Banerjee et al,[11] 2021 | Aghajanian et al,[36] 2022 | Di Silvestro et al,[18] 2023 | Monk et al,[22] 2022 | Gonzalez-Martin et al,[38] 2023 | Li et al,[21] 2023 | Ray-Coquard et al,[12] 2023 | Wu et al,[20] 2024 | Monk et al,[19] 2024 | Gonzalez-Martin et al,[15] 2019 | Moore et al,[39] 2018 |
|---|---|---|---|---|---|---|---|---|---|---|---|---|---|---|
| **randomized trial** | 1b.2 If N/PN/NI to 1b.1: Is it likely that selection of individual participants was affected by knowledge of the intervention assigned to the cluster? | N/A | N/A | N/A | N/A | N/A | N/A | N/A | N/A | N/A | N/A | N/A | N/A | N/A |
| | 1b.3 Were there baseline imbalances that suggest differential identification or recruitment of individual participants between intervention groups? | N | N | N | N | N | N | N | N | N | N | N | N | N |
| | Risk of bias judgement | Low | Low | Low | Low | Low | Low | Low | Low | Low | Low | Low | Low | Low |
| **Domain 2: Risk of bias due to deviations from the intended interventions** | 2.1. Were participants aware of their assigned intervention during the trial? | N | N | N | N | N | N | N | N | N | N | N | N | N |

© 2025 Petousis S et al. *JAMA Network Open*.

| Bias Domain | Signalling Questions | Coleman et al,[37] 2019 | Ray-Coquard et al,[13] 2019 | Banerjee et al,[11] 2021 | Aghajanian et al,[36] 2022 | Di Silvestro et al,[18] 2023 | Monk et al,[22] 2022 | Gonzalez-Martin et al,[38] 2023 | Li et al,[21] 2023 | Ray-Coquard et al,[12] 2023 | Wu et al,[20] 2024 | Monk et al,[19] 2024 | Gonzalez-Martin et al,[15] 2019 | Moore et al,[39] 2018 |
|---|---|---|---|---|---|---|---|---|---|---|---|---|---|---|
| **(effect of assignment to intervention)** | 2.2. Were carers and people delivering the interventions aware of participants' assigned intervention during the trial? | N | N | N | N | N | N | N | N | N | N | N | N | N |
| | 2.3. [If applicable:] If Y/PY/NI to 2.1 or 2.2: Were important non-protocol interventions balanced across intervention groups? | N/A | N/A | N/A | N/A | N/A | N/A | N/A | N/A | N/A | N/A | N/A | N/A | N/A |
| | 2.4. [If applicable:] Were there failures in implementing the intervention that could have affected the outcome? | N | N | N | N | N | N | N | N | N | N | N | N | N |
| | 2.5. [If applicable:] Was there non-adherence to the assigned intervention regimen that could have affected participants' outcomes? | PN | PN | PN | PN | PN | PN | PN | PN | PN | PN | PN | PN | PN |

© 2025 Petousis S et al. *JAMA Network Open*.

| Bias Domain | Signalling Questions | Coleman et al,[37] 2019 | Ray-Coquard et al,[13] 2019 | Banerjee et al,[11] 2021 | Aghajanian et al,[36] 2022 | Di Silvestro et al,[18] 2023 | Monk et al,[22] 2022 | Gonzalez-Martin et al,[38] 2023 | Li et al,[21] 2023 | Ray-Coquard et al,[12] 2023 | Wu et al,[20] 2024 | Monk et al,[19] 2024 | Gonzalez-Martin et al,[15] 2019 | Moore et al,[39] 2018 |
|---|---|---|---|---|---|---|---|---|---|---|---|---|---|---|
| | 2.6. If N/PN/NI to 2.3, or Y/PY/NI to 2.4 or 2.5: Was an appropriate analysis used to estimate the effect of adhering to the intervention? | Y | Y | Y | Y | Y | Y | Y | Y | Y | Y | Y | Y | Y |
| | Risk of bias judgement | Low | Low | Low | Low | Low | Low | Low | Low | Low | Low | Low | Low | Low |
| **Domain 3: Risk of bias due to missing outcome data** | 3.1 Were data for this outcome available for all participants? | Y | Y | Y | Y | Y | Y | Y | Y | Y | Y | Y | Y | Y |
| | 3.2 If N/PN/NI to 3.1: Is there evidence that the result was not biased by missing data? | N/A | N/A | N/A | N/A | N/A | N/A | N/A | N/A | N/A | N/A | N/A | N/A | N/A |
| | 3.3 If N/PN to 3.2 Could missingness in the outcome depend on its true value? | N/A | N/A | N/A | N/A | N/A | N/A | N/A | N/A | N/A | N/A | N/A | N/A | N/A |
| | 3.4 If Y/PY/NI to 3.3: Is it likely that missingness in the outcome depended on | N/A | N/A | N/A | N/A | N/A | N/A | N/A | N/A | N/A | N/A | N/A | N/A | N/A |
| | Risk of bias judgement | Low | Low | Low | Low | Low | Low | Low | Low | Low | Low | Low | Low | Low |

© 2025 Petousis S et al. *JAMA Network Open*.

| Bias Domain | Signalling Questions | Coleman et al,[37] 2019 | Ray-Coquard et al,[13] 2019 | Banerjee et al,[11] 2021 | Aghajanian et al,[36] 2022 | Di Silvestro et al,[18] 2023 | Monk et al,[22] 2022 | Gonzalez-Martin et al,[38] 2023 | Li et al,[21] 2023 | Ray-Coquard et al,[12] 2023 | Wu et al,[20] 2024 | Monk et al,[19] 2024 | Gonzalez-Martin et al,[15] 2019 | Moore et al,[39] 2018 |
|---|---|---|---|---|---|---|---|---|---|---|---|---|---|---|
| **Domain 4: Risk of bias in measurement of the outcome due to missing data** | 4.1 Was the method of measuring the outcome inappropriate? | N | N | N | N | N | N | N | N | N | N | N | N | N |
| | 4.2 Could measurement or ascertainment of the outcome have differed between intervention groups? | N | N | N | N | N | N | N | N | N | N | N | N | N |
| | 4.3a If N/PN/NI to 4.1 and 4.2: Were outcome assessors aware that a trial was taking place? | Y | Y | Y | Y | Y | Y | Y | Y | Y | Y | Y | Y | Y |
| | 4.3b If Y/PY/NI to 4.3a: Were outcome assessors aware of the intervention received by study participants? | N | N | N | N | N | N | N | N | N | N | N | N | N |

© 2025 Petousis S et al. *JAMA Network Open*.

| Bias Domain | Signalling Questions | Coleman et al,[37] 2019 | Ray-Coquard et al,[13] 2019 | Banerjee et al,[11] 2021 | Aghajanian et al,[36] 2022 | Di Silvestro et al,[18] 2023 | Monk et al,[22] 2022 | Gonzalez-Martin et al,[38] 2023 | Li et al,[21] 2023 | Ray-Coquard et al,[12] 2023 | Wu et al,[20] 2024 | Monk et al,[19] 2024 | Gonzalez-Martin et al,[15] 2019 | Moore et al,[39] 2018 |
|---|---|---|---|---|---|---|---|---|---|---|---|---|---|---|
| | 4.4 If Y/PY/NI to 4.3b: Could assessment of the outcome have been influenced by knowledge of intervention received? | N/A | N/A | N/A | N/A | N/A | N/A | N/A | N/A | N/A | N/A | N/A | N/A | N/A |
| | 4.5 If Y/PY/NI to 4.4: Is it likely that assessment of the outcome was influenced by knowledge of intervention received? | N/A | N/A | N/A | N/A | N/A | N/A | N/A | N/A | N/A | N/A | N/A | N/A | N/A |
| | Risk of bias judgement | **Low** | **Low** | **Low** | **Low** | **Low** | **Low** | **Low** | **Low** | **Low** | **Low** | **Low** | **Low** | **Low** |
| **Overall Bias** | Risk of bias judgement | **Low** | **Low** | **Low** | **Low** | **Low** | **Low** | **Low** | **Low** | **Low** | **Low** | **Low** | **Low** | **Low** |

Y=Yes, N=No, PY=Probably Yes, PN=Probably No, N/I=No Information, N/A=Not Applicable

© 2025 Petousis S et al. *JAMA Network Open*.

**eTable 2.** Characteristics of the included studies (PICOS).

| Authors | Year | Population/Patients | Intervention | Comparator | Outcomes | Reported Follow-up (median) | Study (type) |
|---|---|---|---|---|---|---|---|
| Moore et al, [39] (SOLO1) | 2018 | Newly diagnosed, histologically confirmed advanced (FIGO stage III or IV) high-grade serous or endometrioid ovarian, primary peritoneal, and/or fallopian tube cancer with germline or somatic *BRCA1* and/or *BRCA2* mutation on local or central testing. | Olaparib (300 mg twice daily) orally as maintenance monotherapy after standard chemotherapy for up to 2 years or until investigator-assessed objective disease progression, or until treatment was stopped if other discontinuation criteria were met. | Placebo orally as maintenance monotherapy after standard chemotherapy for up to 2 years or until investigator-assessed objective disease progression or treatment was stopped if other discontinuation criteria were met. | Disease progression or death, investigator-assessed PFS HR, median PFS, safety and tolerability, time from randomisation to second disease progression or death, time from randomisation to first and second subsequent treatment or death, and time from randomisation to discontinuation of study treatment or death, whichever occurred first. | 41 months | Prospective randomised, double-blind, placebo-controlled, phase 3 trial. |
| Coleman et al, [37] (VELIA) | 2019 | Women aged 18 years or older with an initial histological diagnosis of high-grade serous epithelial ovarian, fallopian tube, or primary peritoneal cancer at FIGO stage III or IV. | Chemotherapy plus veliparib followed by placebo maintenance (veliparib combination only) or chemotherapy plus veliparib followed by veliparib maintenance (veliparib throughout). | Chemotherapy plus placebo followed by placebo maintenance. | Disease progression or death, PFS HR, median PFS, grade 3-4 adverse event. | 28 months | Prospective randomised, double-blind, placebo-controlled international trial. |
| Ray-Coquard et al, [13] (PAOLA study) | 2019 | Newly diagnosed, advanced, high-grade ovarian cancer having a response after first-line platinum–taxane chemotherapy plus bevacizumab. | Olaparib (300 mg twice daily) at least 3 weeks and no more than 9 weeks after the last dose of chemotherapy up to 24 months or progression AND intravenous bevacizumab in combination with chemotherapy at a dose | Placebo for at least 3 weeks and no more than 9 weeks after the last dose of chemotherapy AND bevacizumab in combination with chemotherapy at a dose of 15 mg per kilogram of body weight every 3 weeks for a total duration of up to 15 months. | Disease progression or death, PFS HR, median PFS, grade 3-4 adverse event. | 22.9 months (range 18.0 - 27.7) | Prospective randomised, double-blind, placebo-controlled international trial. |

© 2025 Petousis S et al. *JAMA Network Open*.

| Authors | Year | Population/Patients | Intervention | Comparator | Outcomes | Reported Follow-up (median) | Study (type) |
|---|---|---|---|---|---|---|---|
| | | | of 15 mg per kilogram of body weight every 3 weeks for a total duration of up to 15 months. | | | | |
| Gonzalez-Martin et al, [15] (PRIMA) | 2019 | Adult patients with newly diagnosed, advanced FIGO stage III/IV, high-grade serous or endometrioid ovarian, primary peritoneal, or fallopian tube cancer who responded to first-line platinum-based chemotherapy. | Within 12 weeks of completion of first-line treatment, patients were randomised 2:1 to receive niraparib orally once daily until progressive disease or intolerable toxicity; patients who were benefitting were eligible to continue receiving treatment beyond the planned 3-year treatment duration. | Within 12 weeks of completion of first-line treatment, patients were randomised 2:1 to receive placebo once daily until progressive disease or intolerable toxicity; patients who were benefitting were eligible to continue receiving treatment beyond the planned 3-year treatment duration. | The primary endpoint was PFS HR and any recurrence/death (first in patients with HRD tumours and then in the overall population), OS, time until the first subsequent therapy, PFS per investigator assessment, safety outcomes, and patient-reported outcomes were secondary end-points. | 24 months | Prospective, phase 3, randomised, double-blind placebo-controlled trial. |
| Banerjee et al, [11] (SOLO1) | 2021 | Newly diagnosed, histologically confirmed advanced (FIGO stage III or IV) high-grade serous or endometrioid ovarian, primary peritoneal, and/or fallopian tube cancer with germline or somatic *BRCA1* and/or *BRCA2* mutation on local or central testing. | Olaparib (300 mg twice daily) orally as maintenance monotherapy after standard chemotherapy for up to 2 years or until investigator-assessed objective disease progression or until treatment was stopped if other discontinuation criteria were met. | Placebo orally as maintenance monotherapy after standard chemotherapy for up to 2 years or until investigator-assessed objective disease progression or treatment was stopped if other discontinuation criteria were met. | Disease progression or death, investigator-assessed PFS HR, median PFS, safety and tolerability, time from randomisation to second disease progression or death, times from randomisation to first and second subsequent therapy or death, and time from randomisation to discontinuation of study treatment or death, whichever occurred first. | 4.8 years (olaparib) and 5.0 years (placebo) | Prospective randomised, double-blind, placebo-controlled, phase 3 trial. |
| Aghajanian et al, [36] (VELIA) | 2022 | Women aged 18 years or older with an initial histological diagnosis of | Chemotherapy plus veliparib followed by placebo maintenance | Chemotherapy plus placebo followed by placebo maintenance. | Disease progression or death, PFS HR, median PFS, grade 3-4 adverse event | 28 months | Prospective randomised, double-blind, |

© 2025 Petousis S et al. *JAMA Network Open*.

| Authors | Year | Population/Patients | Intervention | Comparator | Outcomes | Reported Follow-up (median) | Study (type) |
|---|---|---|---|---|---|---|---|
| | | high-grade serous epithelial ovarian, fallopian tube, or primary peritoneal cancer at FIGO stage III or IV. | (veliparib combination only) or chemotherapy plus veliparib followed by veliparib maintenance (veliparib throughout). | | | | placebo-controlled international trial. Exploratory analyses by paclitaxel dosing schedule and germline *BRCA* status. |
| DiSilvestro et al, [18] (SOLO1) | 2023 | Newly diagnosed, histologically confirmed advanced (FIGO stage III or IV) high-grade serous or endometrioid ovarian, primary peritoneal, and/or fallopian tube cancer with germline or somatic *BRCA1* and/or *BRCA2* mutation on local or central testing. | Olaparib (300 mg twice daily) orally as maintenance monotherapy after standard chemotherapy for up to 2 years or until investigator-assessed objective disease progression or treatment was stopped if other discontinuation criteria were met. | Placebo orally as maintenance monotherapy after standard chemotherapy for up to 2 years or until investigator-assessed objective disease progression or treatment was stopped if other discontinuation criteria were met. | Secondary endpoints reported in this analysis are OS HR, death, time from randomisation to first TFST, TSST, TDT, adverse events. | 88.9 months (olaparib), 87.4 months (placebo) | Prospective randomised, double-blind, placebo-controlled, phase 3 trial (Report after a 7-year follow-up). |
| Monk et al, [22] (ATHENA) | 2022 | Patients aged 18 years or older with newly diagnosed, histologically confirmed, advanced (FIGO stage III-IV), high-grade epithelial ovarian, fallopian tube, or primary peritoneal cancer, with a known BRCA mutation result (either positive or negative) by central testing; ECOG 0-1. | Patients received rucaparib 600 mg orally twice daily starting on day 1 of cycle 1 and placebo every 4 weeks starting on day 1 of cycle 2 in 28-day cycles. Rucaparib treatment could be continued until 24 months after initiation of placebo administration, disease | Patients received placebo orally twice daily starting on day 1 of cycle 1 and placebo every 4 weeks starting on day 1 of cycle 2 in 28-day cycles. Rucaparib treatment could be continued until 24 months after initiation of placebo administration, disease progression, death, or unacceptable toxicity. | Investigator-assessed PFS HR, any progression or death, OS HR, death, investigator-assessed ORR, DOR for patients with investigator-assessed confirmed radiographic complete or partial response, TEAEs. | 26 months | Prospective international, multicenter, randomised, double-blind, phase III trial consisting of four treatment arms. Present study reports comparison of rucaparib maintenance |

© 2025 Petousis S et al. *JAMA Network Open*.

| Authors | Year | Population/Patients | Intervention | Comparator | Outcomes | Reported Follow-up (median) | Study (type) |
|---|---|---|---|---|---|---|---|
| | | | progression, death, or unacceptable toxicity. | | | | treatment versus placebo. |
| Gonzalez-Martin et al, [38] (PRIMA) | 2023 | Adult patients with newly diagnosed, advanced FIGO stage III/IV, high-grade serous or endometrioid ovarian, primary peritoneal, or fallopian tube cancer who have responded to first-line platinum-based chemotherapy. | Within 12 weeks of completion of first-line treatment, patients were randomised 2:1 to receive niraparib orally once daily until progressive disease or intolerable toxicity; patients who benefited were eligible to continue receiving treatment beyond the planned 3-year treatment duration. | Within 12 weeks of completion of first-line treatment, patients were randomised 2:1 to receive placebo once daily until progressive disease or intolerable toxicity; patients who benefited were eligible to continue receiving treatment beyond the planned 3-year treatment duration. | The primary endpoint was PFS HR and any recurrence/death (first in patients with HRD tumours and then in the overall population), PFS by investigator assessment, safety outcomes, and patient-reported outcomes were secondary endpoints. | 36 months | Prospective, phase 3, randomised, double-blind placebo-controlled trial. |
| Li et al, [21] (PRIME) | 2023 | Eligible patients were aged 18 years or older and had a new diagnosis of histologically confirmed, high-grade serous or endometrioid epithelial ovarian cancer, fallopian tube carcinoma or primary peritoneal cancer. | Patients were centrally randomised 2:1 to receive niraparib through an interactive web response system, as stratified by germline *BRCA* variant status (yes or no), tumour HRD status (positive or negative including unknown) on the HRD assay (BGI Genomics), receipt of neoadjuvant chemotherapy (yes or no), and clinical response to treatment with first-line platinum-based chemotherapy (complete or partial) | Patients were centrally randomised 2:1 to receive placebo following first-line platinum-based chemotherapy (complete or partial). | The primary endpoint was BICR-assessed PFS HR, any recurrence or death. The secondary endpoints included OS HR, death, time to first subsequent anticancer therapy | 27 months | Prospective randomised, double-blind, placebo controlled, phase 3 randomized clinical trial. |

© 2025 Petousis S et al. *JAMA Network Open*.

| Authors | Year | Population/Patients | Intervention | Comparator | Outcomes | Reported Follow-up (median) | Study (type) |
|---|---|---|---|---|---|---|---|
| | | | after completion of standard chemotherapy. | | | | |
| Ray-Coquard et al, [12] (PAOLA1) | 2023 | Newly diagnosed, advanced, high-grade ovarian cancer having a response after first-line platinum–taxane chemotherapy plus bevacizumab. | Olaparib (300 mg twice daily) for at least 3 weeks and no more than 9 weeks after the last dose of chemotherapy up to 24 months or progression AND intravenous bevacizumab in combination with chemotherapy at a dose of 15 mg per kilogram of body weight every 3 weeks for a total duration of up to 15 months. | Placebo for at least 3 weeks and no more than 9 weeks after the last dose of chemotherapy AND bevacizumab in combination with chemotherapy at a dose of 15 mg per kilogram of body weight every 3 weeks for a total duration of up to 15 months. | Disease progression or death (updated analysis), PFS HR (updated analysis), median PFS, death, OS HR, grade 3-4 adverse event. | 51 months | Prospective randomised, double-blind, placebo-controlled international trial. |
| Wu et al. [20] (FLAMES) | 2024 | Advanced ovarian cancer FIGO stage III–IV and response to first-line platinum-based chemotherapy. | Senaparib 100 mg orally once daily for up to 2 years after standard chemotherapy. | Placebo orally once daily for up to 2 years after standard chemotherapy. | Disease progression or death, PFS HR, median PFS, death, grade 3-4 adverse event. | 27 months | Prospective multicenter, randomised, double-blinded, phase 3 trial |
| Monk et al, [19] (PRIMA) | 2024 | Adult patients with newly diagnosed, advanced FIGO stage III/IV, high-grade serous or endometrioid ovarian, primary peritoneal, or fallopian tube cancer who responded to first-line platinum-based chemotherapy. | Within 12 weeks of completion of first-line treatment, patients were randomised 2:1 to receive niraparib orally once daily until progressive disease or intolerable toxicity; patients who were benefitting were eligible to continue receiving treatment | Within 12 weeks of completion of first-line treatment, patients were randomised 2:1 to receive placebo once daily until progressive disease or intolerable toxicity; patients who were benefitting were eligible to continue receiving treatment beyond the | The primary endpoint was PFS HR, any recurrence or death. OS HR, any death, TFST, PFS2 (the time from randomisation to the earliest date of assessment of progression on the next anticancer therapy after study treatment or death from any cause), patient-reported adverse events were secondary endpoints. | 43 months | Prospective, double-blind, placebo-controlled phase III (updated, descriptive ad-hoc PFS analysis by investigator assessment). |

© 2025 Petousis S et al. *JAMA Network Open*.

| Authors | Year | Population/Patients | Intervention | Comparator | Outcomes | Reported Follow-up (median) | Study (type) |
|---|---|---|---|---|---|---|---|
| | | | beyond the planned 3-year treatment duration. | planned 3-year treatment duration. | | | |

FIG*O=International Federation of Gynecology and Obstetrics, RCT=Randomized Controlled Trial, PFS=progression-free survival, HR=Hazard ratio, OS=Overall survival, ECOG=Eastern Cooperative Oncology Group, HRD=Homologous recombination deficiency, TFST=Time from randomisation to first subsequent treatment or death, TSST=Time from randomisation to to second subsequent treatment or death, TDT=Time from randomisation to discontinuation of study treatment or death , ORR=Objective response rate, DOR=Duration of response, TEAE=Treatment-emergent adverse events , BICR= Blinded Independent Central Review*

© 2025 Petousis S et al. *JAMA Network Open*.

## Supplementary methods

1. **Abstract/title screening and full-text assessment**
2. **Risk of bias of individual studies**
3. **Overall quality of the evidence**
4. **Descriptive comparative analysis of PARP inhibitor regimens**

### 1. Abstract/title screening and full-text assessment

Upon import into Covidence, further duplicates were manually verified. Subsequently, the titles and abstracts of the search results were independently screened by two reviewers (S.P. and T.A.Z.). Selected references were obtained in full-text and independently assessed for eligibility by two reviewers (S.P. and T.A.Z.). Disagreements over eligibility during title-abstract or full text screening were resolved by a third reviewer (M.F.). To complement the results of bibliographic searching, we contacted experts in the field for potentially missing literature.

### 2. Risk of bias of individual studies

The methodological quality of the included studies was assessed using the RoB 2 tool (Risk Of Bias In Prospective Randomized Studies) [1]. This tool evaluates bias across five key domains: the randomization process, deviations from intended interventions, missing outcome data, measurement of outcomes, and selection of reported results. Each domain was rated as low risk, some concerns, or high risk of bias, with an overall judgment reflecting the highest level of bias observed in any domain.

© 2025 Petousis S et al. *JAMA Network Open*.

The primary outcomes of interest for bias assessment were PFS or OS in all included studies. Assessments were performed independently by two reviewers (S.P. and T.A.Z.), with any disagreements resolved through discussion or consultation with a third reviewer (A.S.).

## 3. Overall quality of the evidence

The overall quality of the evidence for both primary and secondary outcomes was assessed using the GRADE (Grading of Recommendations, Assessment, Development, and Evaluations) framework [2, 3]. This evaluation was conducted with the GRADEpro GDT software. GRADE assesses evidence quality across five domains: RoB, inconsistency, indirectness, imprecision, and publication bias. GRADE specifies four categories for the quality of a body of evidence. The level of confidence decreases with decreasing quality (high → moderate → low → very low) with very low-quality meaning that the true effect is likely to be substantially different from that estimated in the review.

## 4. Descriptive comparative analysis of PARP inhibitor regimens

*Overview*

Due to the absence of direct head-to-head trials comparing different PARP inhibitors, formal network meta-analysis was not performed. Instead, we conducted separate pairwise meta-analyses, each comparing a PARP inhibitor regimen to platinum-based chemotherapy, which served as the common comparator across all included randomized controlled trials. This approach allowed for a descriptive comparison of the relative effects of PARP inhibitors in terms of the incidence of any event, death, and high grade adverse events in the overall population, the HRD subgroup, the *BRCA*-mutated subgroup, the *BRCA* wildtype subgroup, and the HRP subgroup. This approach allowed for the indirect comparison of multiple treatment regimens within a single framework, synthesising a wider range of evidence. Results

© 2025 Petousis S et al. *JAMA Network Open*.

were reported separately for each comparison, without indirect statistical contrasts between PARP inhibitor regimens.

*Geometry of the indirect comparison*

Geometry of indirect comparison was visualized using the netgraph command in the **"netmeta"** package [4] in R (R: a language environment for statistical computing, R Foundation for Statistical Computing, Vienna, Austria), solely to depict the comparative structure of the included trials, not to support a formal network meta-analysis.

*Statistical Analysis*

For each PARP inhibitor, we performed a random-effects pairwise meta-analysis comparing simultaneously its efficacy and safety outcomes to platinum-based chemotherapy [5]. For all pairwise comparisons, RR with 95% CI were estimated using a multivariate meta-analysis approach, which accounts for the correlation introduced by multi-arm trials [6]. Analyses were conducted using the "**netmeta"** package [4] in R, adhering to the intention-to-treat principle.

*Assessment of Inconsistency*

Inconsistency assessment was not possible, as outcomes were based entirely on indirect comparisons.

*Interpretation of Findings*

Comparisons across different PARP inhibitors were interpreted descriptively, based on their respective RRs versus control. No statistical inferences or rankings were made between PARP inhibitors, as such comparisons would require direct or mixed evidence not available in the

© 2025 Petousis S et al. *JAMA Network Open*.

current literature. Surface under the cumulative ranking (SUCRA) analysis and treatment ranking tables were therefore not performed to avoid overinterpretation of purely indirect data.

## References


1. Sterne, J.A.C., et al., *RoB 2: a revised tool for assessing risk of bias in randomised trials.* BMJ, 2019. **366**: p. l4898.
2. Foroutan, F., et al., *GRADE Guidelines 28: Use of GRADE for the assessment of evidence about prognostic factors: rating certainty in identification of groups of patients with different absolute risks.* J Clin Epidemiol, 2020. **121**: p. 62-70.
3. Iorio, A., et al., *Use of GRADE for assessment of evidence about prognosis: rating confidence in estimates of event rates in broad categories of patients.* BMJ, 2015. **350**: p. h870.
4. Rucker, G. and G. Schwarzer, *Ranking treatments in frequentist network meta-analysis works without resampling methods.* BMC Med Res Methodol, 2015. **15**: p. 58.
5. Caldwell, D.M., A.E. Ades, and J.P. Higgins, *Simultaneous comparison of multiple treatments: combining direct and indirect evidence.* BMJ, 2005. **331**(7521): p. 897-900.
6. Salanti, G., *Indirect and mixed-treatment comparison, network, or multiple-treatments meta-analysis: many names, many benefits, many concerns for the next generation evidence synthesis tool.* Res Synth Methods, 2012. **3**(2): p. 80-97.


© 2025 Petousis S et al. *JAMA Network Open*.

# Search strategies

## Embase.com

(20240820; 2,305 hits)

('adnexal tumor (gynecologic)'/de OR 'adnexal cancer (gynecologic)'/de OR 'ovary tumor'/de OR 'ovary cancer'/de OR 'ovary carcinoma'/exp OR 'ovarian epithelial tumor'/de OR 'high grade serous ovarian cancer'/de OR 'high grade serous ovarian carcinoma'/de OR 'high grade serous ovary cancer'/de OR 'high grade serous ovary carcinoma'/de OR 'high grade serous carcinoma'/de OR 'fallopian tube tumor'/exp OR 'peritoneum tumor'/de OR 'peritoneum cancer'/de OR 'primary peritoneal carcinoma'/de OR 'cystadenocarcinoma'/de OR (((ovar* OR tuboovar* OR tube* OR tubal OR peritoneal OR peritoneum OR adnex* OR oviduct) NEAR/4 (carcino* OR cancer* OR neoplas* OR tumor* OR tumour* OR malignan* OR metastas* OR adenocarcino*)) OR cystadenocarcinoma):ab,ti,kw)

AND

('maintenance therapy'/de OR 'maintenance chemotherapy'/exp OR (maintenance OR ((long-term OR longterm OR chronic) NEAR/3 (therapy OR therapies OR chemotherapy OR chemotherapies OR carcinochemotherapy OR carcinochemotherapies OR treatment*))):ab,ti,kw)

AND

('nicotinamide adenine dinucleotide adenosine diphosphate ribosyltransferase inhibitor'/exp OR ('NAD ADP ribosyltransferase inhibitor*' OR 'PARP inhibitor*' OR 'PARP-1 inhibitor*' OR 'PARS inhibitor*' OR 'poly ADP ribose polymerase inhibitor*' OR 'poly ADP ribose synthetase inhibitor*' OR PARPi OR 'meta aminobenzamide' OR '3 aminobenzamide' OR '3 methoxybenzamide' OR '3 nitrosobenzamide' OR amelparib OR atamparib OR basroparib OR airuiyi OR cofpropamine OR fluzoparib OR fuzuloparib OR iniparib OR lerzeparib OR letermovir OR nesuparib OR zejula OR niraparib OR olaparib OR pamiparib OR phenanthridone OR rubraca OR rucaparib OR saruparib OR senaparib OR talzenna OR talazoparib OR veliparib OR 'TNKS inhibitor*' OR 'tankyrase inhibitor*'):ab,ti,kw)

NOT

(('animal'/de OR 'animal experiment'/exp OR 'nonhuman'/de) NOT ('human'/exp OR 'human experiment'/de))

## Medline Ovid

(20240820; Ovid MEDLINE(R) ALL 1946 to August 19, 2024; 849 hits)

("ovarian neoplasms"/ or "carcinoma, ovarian epithelial"/ or "fallopian tube neoplasms"/ or "peritoneal neoplasms"/ OR (((ovar* OR tuboovar* OR tube* OR tubal OR peritoneal OR peritoneum OR adnex* OR oviduct) ADJ4 (carcino* OR cancer* OR neoplas* OR tumor* OR tumour* OR malignan* OR metastas* OR adenocarcino*)) OR cystadenocarcinoma).ab,ti,kf.)

AND

(maintenance chemotherapy/ OR (maintenance OR ((long-term OR longterm OR chronic) ADJ3 (therapy OR therapies OR chemotherapy OR chemotherapies OR carcinochemotherapy OR carcinochemotherapies OR treatment*))).ab,ti,kf.)

© 2025 Petousis S et al. *JAMA Network Open*.

AND

("poly(adp-ribose) polymerase inhibitors"/ OR ("3-methoxybenzamide" OR "3-aminobenzamide" OR "phenanthridone" OR "6-amino-1,2-benzopyrone" OR "6-nitroso-1,2-benzopyrone" OR "3-nitrosobenzamide" OR "4-amino-1,8-naphthalimide" OR "iniparib" OR "1,5-dihydroxyisoquinoline" OR "cofpropamine" OR "3,4-dihydro-5-(4-(1-piperidinyl)butoxy)-1(2H)-isoquinolinone" OR "2-(4-hydroxyphenyl)-1H-benzimidazole-4-carboxamide" OR "1,5-dihydroisoquinoline" OR "thieno(2,3-c)isoquinolin-5-one" OR "3,4-dihydro-5-hydroxy-1(2H)-isoquinolinone" OR "2-(4-cyclopropylmethylpiperazin-1-yl)-5H-benzo(c)(1,5)naphthyridin-6-one" OR "2-(4-chlorophenyl)-5-quinoxalinecarboxamide" OR "GPI 15427" OR "2-((2-piperidin-1-ylethyl)thio)quinazolin-4(3H)-one" OR "KU0058948" OR "veliparib" OR "rucaparib" OR "olaparib" OR "4-(3-(4-cyclopropanecarbonylpiperazine-1-carbonyl)-4-fluorobenzyl)-2H-phthalazin-1-one" OR "2-(methoxycarbonyl(4-methoxyphenyl)methylsulfanyl)-1H-benzimidazole-4-carboxylic acid amide" OR "2-(4-(pyridin-2-yl)phenyl)-1H-benzo(d)imidazole-4-carboxamide" OR "niraparib" OR "2-(2-fluoro-4-(pyrrolidin-2-yl)phenyl)-1H-benzimidazole-4-carboxamide" OR "10-((4-hydroxypiperidin-1-yl)methyl)chromeno(4,3,2-de)phthalazin-3(2H)-one" OR "talazoparib" OR "ME0328" OR "letermovir" OR "pamiparib" OR "PARP-1 inhibitor DHC-1" OR "fluzoparib").nm. OR (NAD ADP ribosyltransferase inhibitor* OR PARP inhibitor* OR PARP-1 inhibitor* OR PARS inhibitor* OR poly ADP ribose polymerase inhibitor* OR poly ADP ribose synthetase inhibitor* OR PARPi OR meta aminobenzamide OR 3 aminobenzamide OR 3 methoxybenzamide OR 3 nitrosobenzamide OR amelparib OR atamparib OR basroparib OR airuiyi OR cofpropamine OR fluzoparib OR fuzuloparib OR iniparib OR lerzeparib OR letermovir OR nesuparib OR zejula OR niraparib OR olaparib OR pamiparib OR phenanthridone OR rubraca OR rucaparib OR saruparib OR senaparib OR talzenna OR talazoparib OR veliparib OR TNKS inhibitor* OR tankyrase inhibitor*).ab,ti,kf.)

NOT (exp animals/ NOT humans/)

## Web of Science Core Collection

(20240820; Web of Science Core Collection, Editions = A&HCI , BKCI-SSH , BKCI-S , CCR-EXPANDED , ESCI , IC , CPCI-SSH , CPCI-S , SCI-EXPANDED , SSCI; 1,969 hits)

TS=((((ovar* OR tuboovar* OR tube* OR tubal OR peritoneal OR peritoneum OR adnex* OR oviduct) NEAR/4 (carcino* OR cancer* OR neoplas* OR tumor* OR tumour* OR malignan* OR metastas* OR adenocarcino*)) OR cystadenocarcinoma)

AND

(maintenance OR ((long-term OR longterm OR chronic) NEAR/2 (therapy OR therapies OR chemotherapy OR chemotherapies OR carcinochemotherapy OR carcinochemotherapies OR treatment*)))

AND

("NAD ADP ribosyltransferase inhibitor*" OR "PARP inhibitor*" OR "PARP-1 inhibitor*" OR "PARS inhibitor*" OR "poly ADP ribose polymerase inhibitor*" OR "poly ADP ribose synthetase inhibitor*" OR PARPi OR "meta aminobenzamide" OR "3 aminobenzamide" OR "3 methoxybenzamide" OR "3 nitrosobenzamide" OR amelparib OR atamparib OR basroparib OR airuiyi OR cofpropamine OR fluzoparib OR fuzuloparib OR iniparib OR lerzeparib OR letermovir OR nesuparib OR zejula OR niraparib OR olaparib OR pamiparib OR phenanthridone OR rubraca OR rucaparib OR saruparib OR senaparib OR talzenna OR talazoparib OR veliparib OR "TNKS inhibitor*" OR "tankyrase inhibitor*"))

© 2025 Petousis S et al. *JAMA Network Open*.

## CENTRAL

(20240820; Cochrane Central Register of Controlled Trials, Issue 7 of 12, July 2024; 607 hits)

(((ovar* OR tuboovar* OR tube* OR tubal OR peritoneal OR peritoneum OR adnex* OR oviduct) NEAR/4 (carcino* OR cancer* OR neoplas* OR tumor* OR tumour* OR malignan* OR metastas* OR adenocarcino*)) OR cystadenocarcinoma):ab,ti,kw

AND

(maintenance OR ((long-term OR longterm OR chronic) NEAR/3 (therapy OR therapies OR chemotherapy OR chemotherapies OR carcinochemotherapy OR carcinochemotherapies OR treatment*))):ab,ti,kw

AND

('NAD ADP ribosyltransferase inhibitor*' OR 'PARP inhibitor*' OR 'PARP-1 inhibitor*' OR 'PARS inhibitor*' OR 'poly ADP ribose polymerase inhibitor*' OR 'poly ADP ribose synthetase inhibitor*' OR PARPi OR 'meta aminobenzamide' OR '3 aminobenzamide' OR '3 methoxybenzamide' OR '3 nitrosobenzamide' OR amelparib OR atamparib OR basroparib OR airuiyi OR cofpropamine OR fluzoparib OR fuzuloparib OR iniparib OR lerzeparib OR letermovir OR nesuparib OR zejula OR niraparib OR olaparib OR pamiparib OR phenanthridone OR rubraca OR rucaparib OR saruparib OR senaparib OR talzenna OR talazoparib OR veliparib OR 'TNKS inhibitor*' OR 'tankyrase inhibitor*'):ab,ti,kw

## Clinicaltrials.gov

(20240820; 343 hits)

**Condition/disease:**

Ovary cancer

**Intervention/treatment:**

PARP Inhibitors OR PARPi OR Poly ADP-Ribose Polymerase Inhibitors OR Niraparib OR Olaparib OR fluzoparib OR fuzuloparib OR iniparib OR pamiparib OR rucaparib OR senaparib OR talazoparib OR veliparib

## Search narrative

- By including the textword "maintenance", the second search concept on maintenance chemotherapy was designed sensitively. Nevertheless, this might not catch all relevant abstracts (unless these are indexed with relevant subject headings), e.g., when author only stated something like "patients were treated with PARPi after primary chemotherapy" or "progression to PARPi" or similar. This should be acknowledged as a limitation of the search.

© 2025 Petousis S et al. *JAMA Network Open*.
