# Supplementary material for: PARP Inhibitor Maintenance After First-Line Chemotherapy in Advanced-Stage Epithelial Ovarian Cancer: A Systematic Review and Meta-Analysis
Source: JAMA Netw Open. 2025 Nov 5;8(11):e2541648. doi: 10.1001/jamanetworkopen.2025.41648 (PMC12590296; doi:10.1001/jamanetworkopen.2025.41648)
Supplement: Supplement 2. — Data Sharing Statement [file jamanetwopen-e2541648-s002.pdf]

Data Sharing Statement

Petousis. PARP Inhibitor Maintenance After First-Line Chemotherapy in Advanced-Stage Epithelial Ovarian Cancer: A Systematic Review and Meta-Analysis. JAMA Netw Open. Published online November 5, 2025. doi:10.1001/jamanetworkopen.2025.41648

Data
Data available: Yes
Data types: Other (please specify)
Additional Information: All data used in this systematic review and meta-analysis were obtained from publicly available sources or published studies.
How to access data: All data used in this systematic review and meta-analysis were obtained from publicly available sources or published studies.
When available: With publication

Supporting Documents
Document types: None

Additional Information
Who can access the data: Anyone requesting the data.
Types of analyses: Any purpose.
Mechanisms of data availability: All data used in this systematic review and meta-analysis were obtained from publicly available sources or published studies.
Any additional restrictions: No restriction.
